# Synthesis and crystal structure of a one-dimensional chain-like strontium(II) coordination polymer built of *N*-methyldi­ethano­lamine and isobutyrate ligands

**DOI:** 10.1107/S2056989021005594

**Published:** 2021-06-11

**Authors:** Maximilian Seiss, Sebastian Schmitz, Martin Börner, Kirill Yu. Monakhov

**Affiliations:** a Leibniz Institute of Surface Engineering (IOM), Permoserstr. 15, 04318 Leipzig, Germany

**Keywords:** crystal structure, strontium, carboxyl­ates, *N*-methyldi­ethano­lamine, coordination polymer, zirconium

## Abstract

The one-dimensional coordination polymer (**I**) [Sr(ib)_2_(H_2_mda)]_*n*_ (Hib = isobutyric acid, C_4_H_8_O_2_, and H_2_mda = *N*-methyldi­ethano­lamine, C_5_H_13_NO_2_), namely, *catena*-poly[[(*N*-methyldi­ethano­lamine-κ^3^ *O*,*N*,*O*′)strontium(II)]-di-μ_2_-isobutyrato-κ^3^ *O*,*O*′:*O*;κ^3^ *O*:*O*,*O*′], was prepared by the one-pot aerobic reaction of [Zr_6_O_4_(OH)_4_(ib)_12_(H_2_O)]·3Hib with Sr(NO_3_)_2_ and H_2_mda in the presence of MnCl_2_ and Et_3_N in aceto­nitrile. The use of MnCl_2_ is key to the isolation of **I** as high-quality colorless crystals in good yield. The mol­ecular solid-state structure of **I** was determined by single-crystal X-ray diffraction. Compound **I** crystallizes in the monoclinic space group *P*2_1_/*c* and shows a one-dimensional polymeric chain structure. Each monomeric unit of this coordination polymer consists of a central Sr^II^ ion in the NO_8_ coordination environment of two deprotonated ib^−^ ligands and one fully protonated H_2_mda ligand. The C and O atoms of the H_2_mda ligand were refined as disordered over two sets of sites with site occupancies of 0.619 (3) and 0.381 (3). Compound **I** shows thermal stability up to 130°C in air.

## Abstract 2

The structure of the product obtained from the reaction of [Zr_6_O_4_(OH)_4_(ib)_12_(H_2_O)]·3Hib (Hib = isobutyric acid) with Sr(NO_3_)_2_ and H_2_mda (H_2_mda=*N-*methyldi­ethano­lamine) in the presence of MnCl_2_ and Et_3_N in aceto­nitrile was shown to be the one-dimensional coordination polymer [Sr(ib)_2_(H_2_mda)]_*n*_. Although manganese did not incorporate into the structure, its addition was the key to the isolation of a high-quality crystalline product.

## Chemical context   

Simple metal isobutyrate salts such as *TM*(ib)_2_ (*e.g. TM* = Mn, Co and Ni; Hib = isobutyric acid) and *AM*(ib) (*e.g. AM* = Na and K) are known to act as precursor materials for the synthesis of a wide variety of polynuclear coordination com­plexes, *e.g.* [Mn^II^_4_Mn^III^_2_(ib)_8_(Hbda)_2_(bda)_2_] (H_2_bda = *N*-but­yl­diethano­lamine), [Mn^II^_4_Co^III^_2_(ib)_8_(Hmda)_2_(mda)_2_] (Malae­stean *et al.*, 2010 ▸), [Co^II^_3_Co^III^_2_(Hbda)_2_(bda)_2_(ib)_6_]·2MeCN and [Ni^II^_4_(Hbda)_3_(ib)_5_(MeCN)] (Schmitz *et al.*, 2016 ▸), [Gd^III^_4_ *M* ^II^_8_(OH)_8_(Lig)_8_(ib)_8_](ClO_4_)_4_ (*M* = Zn^II^ or Cu^II^, HLig = 2-(hy­droxy­meth­yl)pyridine); Hooper *et al.*, 2012 ▸) and [Cr_3_O(ib)_6_(H_2_O)_3_](NO_3_) (Parsons *et al.*, 2000 ▸). The formation of these polynuclear homo- and heterometallic complexes was enabled by the introduction of flexible amino alcohol ligands into the reaction mixtures (Schmitz *et al.*, 2016 ▸; Malaestean *et al.*, 2010 ▸). We describe here the first example of a coordination polymer composed of monomeric Sr^II^ units that are supported by both isobutyrate and amino alcohol ligands. This makes the synthesized compound [Sr(ib)_2_(H_2_mda)]_*n*_ (**I**) an appealing precursor for reactions with transition metal and lanthanide complexes. In addition, **I** can find application in solvothermal reactions as it is described, *e.g.* for the transformation of [Co^II^_3_Co^III^_2_(Hbda)_2_(bda)_2_(ib)_6_] to [Co^II^_10_(OH)_2_(bda)_6_(ib)_6_] (Schmitz *et al.*, 2018 ▸). Herein compound **I** was isolated as colorless crystals from an aerobic reaction, characterized by infrared (IR) spectroscopy, thermogravimetric analysis (TGA) and single-crystal X-ray diffraction. Compound **I** represents a rare class of alkaline earth metal–isobutyrate complexes with a 1D polymeric structure (*cf.* {[Mg(ib)_2_(H_2_O)_3_]·H_2_O}_*n*_ (Malaestean *et al.*, 2013 ▸)). Remarkably, MnCl_2_ is crucial in the synthesis of **I** for the formation of high-quality single crystals (in 36% yield) suitable for X-ray diffraction. When the reaction is carried out without MnCl_2_, poor quality crystalline material is formed in lower yield within several days. For the syntheses of homometallic coordination complexes it is common to use an additional metal salt, which yields a heterometallic reaction mixture, from which the homometallic complex can be obtained selectively as a solid product (Ako *et al.*, 2007 ▸; Liu *et al.*, 2018 ▸). In 2007, Ako and co-workers described two hepta­nuclear iron(III) complexes [Fe^III^_7_O_3_(bda)_3_(piv)_9_(H_2_O)_3_] and [Fe^III^_7_O_3_(phda)_3_(piv)_9_(H_2_O)_3_] (H_2_phda = *N*-phenyldi­ethano­lamine and Hpiv = pivalic acid), which were obtained by the reaction of [Fe_3_O(piv)_6_]piv, nickel(II) acetate tetra­hydrate (Ni(OAc)_2_·4H_2_O) and H_2_bda or H_2_phda in a molar ratio of 1:1:2 using MeCN as solvent (Ako *et al.*, 2007 ▸). Although Ni(OAc)_2_·4H_2_O was used in an equimolar ratio with the iron(III) precursor, nickel did not incorporate into the final product. Similar to this, Liu *et al.* (2018 ▸) synthesized a hexa­nuclear [Zn_6_(Lig)_6_(OOCH)_6_] complex (HLig = 4′-(4-carb­oxy­phen­yl)-2,2′:6′,2′′-terpyridine) by the reaction of zinc(II) acetate, Zn(OAc)_2_, with HLig in the presence of praseodymium(III) nitrate hexa­hydrate, Pr(NO_3_)_3_·6H_2_O, using a 2:2:1 molar ratio. The reaction was performed solvothermally in DMF and praseodymium did not incorporate into the final [Zn_6_(Lig)_6_(OOCH)_6_] complex, which was isolated as a pure product by filtration (Liu *et al.*, 2018 ▸). Here [Sr(ib)_2_(H_2_mda)]_*n*_ was also isolated as a pure product by filtration, which indicates that the additional metal salts (here MnCl_2_) remain in the mother liquor.
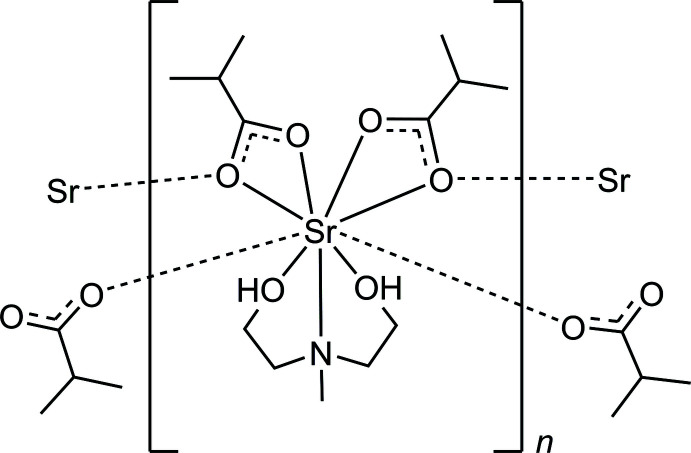


## Structural commentary   

**Figure 1:**
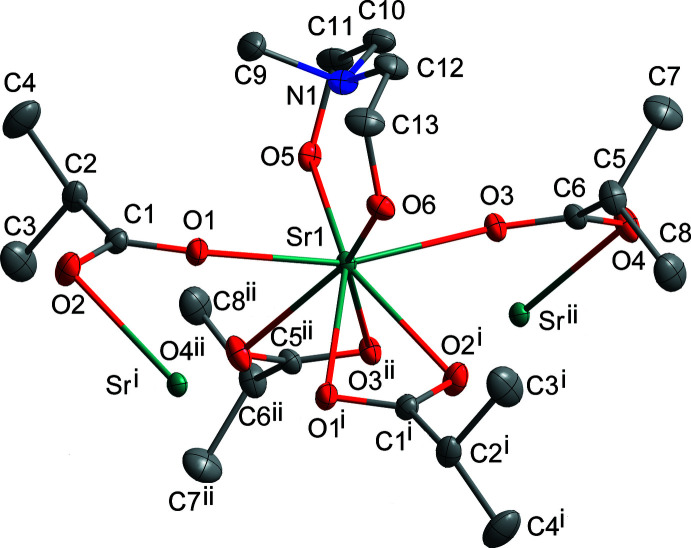
Ellipsoid plot of the monomeric unit of **I** with displacement ellipsoids at the 30% probability level for all non-H atoms. H atoms are omitted for clarity. Color code: Sr teal, C gray, N blue, O red. Disordered atoms are omitted for clarity. Symmetry codes: (i) 2 − *x*, 1 − *y*, 1 − *z*; (ii) 1 − *x*, 1 − *y*, 1 − *z*.

**Figure 2:**
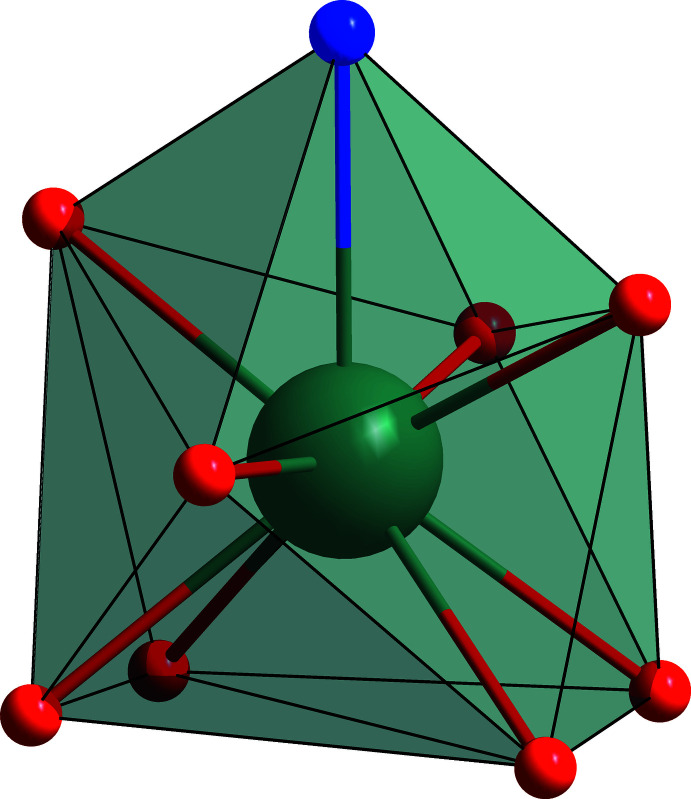
Representation of a polyhedron around a central Sr ion spanned by the NO_8_ coordination environment. Color code: Sr teal, N blue, O red, polyhedron borders black and polyhedron faces transparent.

The crystal structure consists of a Sr^II^ monomer unit (Fig. 1 ▸) extending along the *a-*axis direction. The asymmetric unit contains one central Sr^II^ ion, which is coordinated by a disordered, tridentate and fully protonated H_2_mda and two deprotonated isobutyrate ligands. In other words, Sr^II^ is nine-coordinated by six O atoms (O1, O3, O1^i^, O3^ii^, O2^i^, and O4^ii^; see Table 1 ▸ for geometric parameters and symmetry codes) from four different carboxyl­ate groups, two O atoms (O5 and O6 or O5*A* and O6*A*) and one N atom (N1) from the *N*-methyldi­ethano­lamine ligand. The resulting coordination environment of the strontium center is NO_8_. The polyhedral shape of Sr was evaluated using the *SHAPE* software version 2.1 (Llunell *et al.*, 2013 ▸) and can be described as an in-between a distorted spherical capped square anti­prism and a distorted spherical tricapped trigonal prism (Fig. 2 ▸). The values of the deviation from the ideal geometry are listed in Table 2 ▸. The Sr—O_ib_ bond lengths of the bridging O atoms are between 2.5377 (10) and 2.7563 (10) Å, whereas the non-bridging Sr—O_ib_ bond lengths range from 2.6270 (11) to 2.6364 (11) Å. The non-bonding Sr⋯Sr distances are 4.2869 (3) and 4.2982 (3) Å with Sr—O—Sr angles of 108.50 (4) and 108.84 (5)°. The Sr—O_H2mda_ bond lengths range between 2.582 (20) and 2.731 (11) Å, and Sr—N bond length is 2.8495 (13) Å.

## Supra­molecular features   

**Figure 3:**
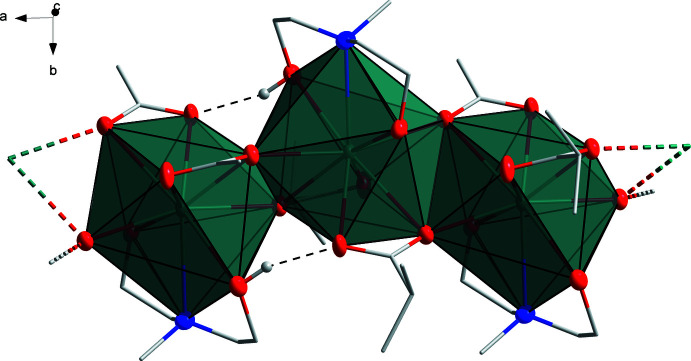
Representation of a segment of the polymeric structure of [Sr(ib)_2_(H_2_mda)]_*n*_ (**I**) along the crystallographic *c* axis. Color code: Sr teal, C gray, N blue, O red, bridging O spheres red and H atoms white. Hydrogen bonds are shown as dashed black lines. Disordered fragments are omitted for clarity.

The crystal packing reveals the existence of 1D polymeric zigzag chains running along the *a-*axis direction (Figs. 3 ▸ and 4 ▸), in which monomeric Sr^II^ units are inter­linked by one O atom of each isobutyrate ligand, which are all coordinated in a chelating, bridging μ_2_-η^2^:η^1^ mode. The H_2_mda ligands coord­inate in the chelating μ_1_-η^1^: η^1^: η^1^ mode to the Sr centers of **I**. The edge-sharing SrNO_8_ polyhedra are linked by the isobutyrate O1 and O1^ii^ atoms on the one side and O3 and O3^i^ atoms on the other side. Intra­molecular hydrogen bonding is present along the chains *via* O5—H5⋯O2, O6—H6⋯O4 and O6*A*—H6*A*⋯O4 contacts (Fig. 3 ▸, Table 3 ▸).

## Database survey   

A search of the Cambridge Structural Database (CSD, version 5.42, update of November 2020; Groom *et al.*, 2016 ▸) resulted in 34 hits for metal complexes ligated by isobutyrate and *N*-alkyl­diethano­lamine. To the best of our knowledge, there are no alkaline earth complexes as well as coordination polymers incorporating both ligands. There are four polymeric structures solely containing group two elements and isobutyrate anions: the magnesium complex *catena*-poly[[tri­aqua­(isobutyrato)-κ*O*)magnesium]-μ-isobutyrato-κ^2^ *O:O′*] monohydrate, refcode VIQTOG (Malaestean *et al.*, 2013 ▸), *catena*-poly[[μ-aqua-di­aqua­(μ_3_-2-methyl­propano­ato-κ^4^ *O:O,O′:O′*)calcium] 2-methyl­propano­ate dihydrate], refcode JUWMEW (Samolová & Fábry, 2020 ▸), as well as the isostructural strontium complex, refcode JUWMIA (Samolová & Fábry, 2020 ▸) and the mixed calcium/strontium complex *catena*-poly[[μ-aqua-di­aqua­(μ_3_-2-methyl­propano­ato-κ^4^ *O:O,O′:O′*)calcium/strontium] 2-methyl­propano­ate dihydrate], refcode JUWMOG (Samolová & Fábry, 2020 ▸).

## Synthesis and crystallization   

**Figure 5:**
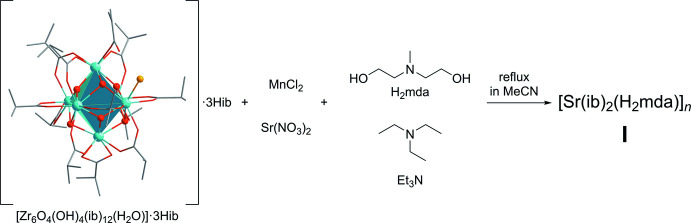
Synthesis of compound **I**.

The one-pot reaction of freshly prepared hexa­nuclear zirconium complex [Zr_6_O_4_(OH)_4_(ib)_12_(H_2_O)]·3Hib (Kogler *et al.*, 2004 ▸, abbreviated as {Zr_6_}) with strontium(II) nitrate and manganese(II) chloride in a 1.0:2.2:2.2, molar ratio was performed in aceto­nitrile under aerobic conditions, involving 11.1 eq. of *N*-methyldi­ethano­lamine as a co-ligand and 4.0 eq. of tri­ethyl­amine as a base (see Fig. 5 ▸). The polymeric coord­ination complex [Sr(ib)_2_(H_2_mda)]_*n*_ (**I**) was isolated as colorless crystals. By-products could not be identified. The IR spectrum of **I** is characterized by the asymmetric O–C–O vibration bands at 1556 cm^−1^ and the symmetric O–C–O ones in the range of 1366–1426 cm^−1^.

**Figure 6:**
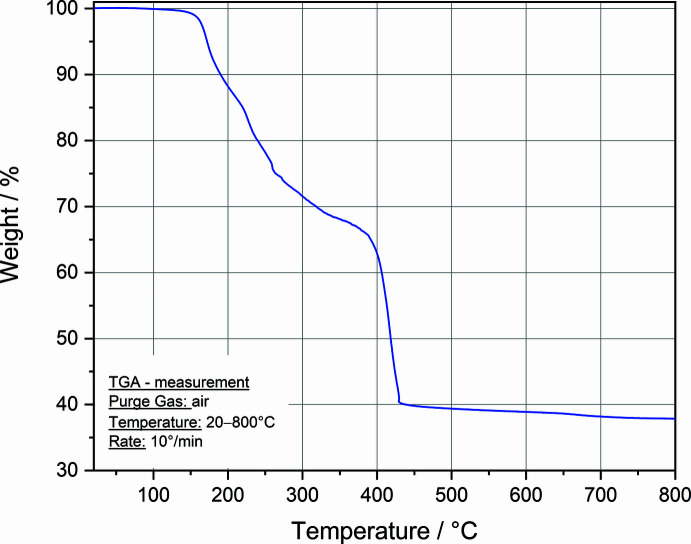
Thermogravimetric analysis for **I**.

The TGA curve (Fig. 6 ▸) shows that the thermal decomposition of **I** occurs between 130 and 440°C with a mass loss of C_12_H_27_NO_3_ per monomer unit (Δ*m*_total_ = 60.00% *vs* Δ*m*_calcd._ = 61.25%), and it yields SrCO_3_. Overall, the thermal stability of **I** up to 130°C in air is similar to that determined for isobutyrate di­ethano­lamine complexes of cobalt (140°C) and nickel (130°C) (Schmitz *et al.*, 2016 ▸).

## Refinement   

Crystal data, data collection and structure refinement details are summarized in Table 4 ▸. The structure was solved using dual space methods and refined by full-matrix least-squares minimization on *F^2^*. The coordinates of all non-hydrogen atoms were refined with anisotropic thermal parameters. All H atoms were placed in geometrically idealized positions and refined using a rigid model and included as riding atoms, with methyl C—H = 0.98 Å, methyl­ene C—H = 0.99 Å, methine C—H = 1.00 Å and O—H = 0.84 Å. Isotropic displacement parameters were set to *U*_iso_(H) = 1.2*U*_eq_ for the parent atom (1.5 for methyl and hy­droxy groups). The hy­droxy groups and the idealized methyl group were refined as rotating. Atoms C9, C10, C11, C12, C13, O5 and O6 of the H_2_mda ligand were refined as disordered over two sets of sites with site occupancies of 0.619 (3) and 0.381 (3). As a result of the short distance between the disordered atoms C11, C13, O5, O6 and their corresponding counterparts, EADP constraints were applied to equalize the displacement ellipsoids of the atom pairs.

## Supplementary Material

Crystal structure: contains datablock(s) I. DOI: 10.1107/S2056989021005594/dj2022sup1.cif

Structure factors: contains datablock(s) I. DOI: 10.1107/S2056989021005594/dj2022Isup3.hkl

Supporting information file. DOI: 10.1107/S2056989021005594/dj2022sup4.zip

CCDC reference: 2087088

Additional supporting information:  crystallographic information; 3D view; checkCIF report

## Figures and Tables

**Figure 4:**
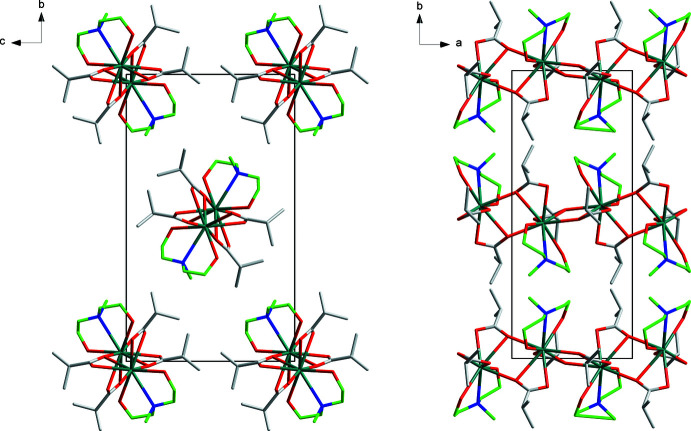
Packing diagram of **I**, viewed down the *a* axis (left) and the *c* axis (right). Color code: Sr teal, C (ib) gray, C (H_2_mda) green, N blue, O red. H atoms and disordered fragments are omitted for clarity.

**Table 1:** Selected geometric parameters (Å, °)

Sr1—O1^i^	2.7563 (10)	Sr1—O5	2.731 (11)
Sr1—O3^ii^	2.7244 (11)	Sr1—O6	2.66 (3)
Sr1—O1	2.5377 (10)	Sr1—N1	2.8495 (13)
Sr1—O3	2.5444 (10)	Sr1⋯Sr1^i^	4.2981 (3)
Sr1—O2^i^	2.6270 (11)	Sr1⋯Sr1^ii^	4.2868 (3)
Sr1—O4^ii^	2.6364 (11)		
			
Sr1—O1—Sr1^i^	108.50 (4)	Sr1—O1—Sr1^ii^	108.84 (5)

Symmetry codes: (i) -x+2, -y+1, -z+1; (ii) -x+1, -y+1, -z+1.

**Table 2:** Selected Continuous Shapes Measures (CShM) values for the geometry about the nine-coordinate Sr^II^ ions of **I**

Shape	Capped square anti­prism (*C*_4v_, J10)	Spherical capped square anti­prism (*C*_4v_)	Tricapped trigonal prism (*D*_3h_, J51)	Spherical tricapped trigonal prism (*D*_3h_)	Muffin (*C*_s_)
Sr^i^	4.349	3.765	5.892	3.696	3.732
Sr^ii^	4.026	3.346	5.575	3.423	3.358

Symmetry codes: (i) -x+2, -y+1, -z+1; (ii) -x+1, -y+1, -z+1.

**Table 3:** Hydrogen-bond geometry (Å, °)

*D*—H⋯*A*	*D*—H	H⋯*A*	*D*⋯*A*	*D*—H⋯*A*
O6*A*—H6*A*⋯O4^iii^	0.84	1.83	2.61 (5)	153
O6—H6*B*⋯O4^iii^	0.84	1.95	2.75 (3)	160
O5—H5⋯O2^iv^	0.84	1.87	2.680 (12)	163

Symmetry codes: (iii) x+1, y, z; (iv) x-1, y, z.

**Table 4:** Experimental details

Crystal data	
Chemical formula	[Sr(C_4_H_7_O_2_)_2_(C_5_H_13_NO_2_)]
*M*_r_	380.97
Crystal system, space group	Monoclinic, *P*2_1_/*c*
Temperature (K)	180
*a*, *b*, *c* (Å)	8.1516 (2), 19.1921 (6), 11.4288 (3)
β (°)	99.295 (2)
*V* (Å^3^)	1764.52 (8)
*Z*	4
Radiation type	Cu *K*α
μ (mm^−1^)	4.46
Crystal size (mm)	0.28 × 0.21 × 0.13

Data collection	
Diffractometer	Stoe Stadivari
Absorption correction	Multi-scan (*X-AREA* *LANA*; Stoe, 2019 ▸)
*T*_min_, *T*_max_	0.178, 0.458
No. of measured, independent and observed [*I* > 2σ(*I*)] reflections	15496, 3300, 3009
*R*_int_	0.014
(sin θ/λ)_max_ (Å^−1^)	0.611

Refinement	
*R*[*F*^2^ > 2σ(*F* ^2^)], *wR*(*F* ^2^), *S*	0.018, 0.046, 1.06
No. of reflections	3300
No. of parameters	240
H-atom treatment	H-atom parameters constrained
Δρ_max_, Δρ_min_ (e Å^−3^)	0.39, −0.19

Computer programs: *X-AREA Pilatus3_SV*, * Recipe* and *Integrate* (Stoe, 2019 ▸), *olex2.solve* (Bourhis *et al.*, 2015 ▸), *SHELXL2018/3* (Sheldrick, 2015 ▸) and *OLEX2* (Dolomanov *et al.*, 2009 ▸).

## supplementary crystallographic information

### Crystal data

**Table:**

[Sr(C_4_H_7_O_2_)_2_(C_5_H_13_NO_2_)]	*F*(000) = 792
*M**_r_* = 380.97	*D*_x_ = 1.434 Mg m^−^^3^
Monoclinic, *P*2_1_/*c*	Cu *K*α radiation, λ = 1.54186 Å
*a* = 8.1516 (2) Å	Cell parameters from 16191 reflections
*b* = 19.1921 (6) Å	θ = 4.5–70.9°
*c* = 11.4288 (3) Å	µ = 4.46 mm^−^^1^
β = 99.295 (2)°	*T* = 180 K
*V* = 1764.52 (8) Å^3^	Block, light yellow
*Z* = 4	0.28 × 0.21 × 0.13 mm

### Data collection

**Table:**

Stoe Stadivari diffractometer	3300 independent reflections
Radiation source: GeniX 3D HF Cu	3009 reflections with *I* > 2σ(*I*)
Graded multilayer mirror monochromator	*R*_int_ = 0.014
Detector resolution: 5.81 pixels mm^-1^	θ_max_ = 70.5°, θ_min_ = 4.6°
rotation method, ω scans	*h* = −6→9
Absorption correction: multi-scan (XAREA *LANA*; Stoe, 2019)	*k* = −23→22
*T*_min_ = 0.178, *T*_max_ = 0.458	*l* = −13→13
15496 measured reflections	

### Refinement

**Table:**

Refinement on *F*^2^	Primary atom site location: iterative
Least-squares matrix: full	Hydrogen site location: inferred from neighbouring sites
*R*[*F*^2^ > 2σ(*F*^2^)] = 0.018	H-atom parameters constrained
*wR*(*F*^2^) = 0.046	*w* = 1/[σ^2^(*F*_o_^2^) + (0.031*P*)^2^ + 0.1664*P*] where *P* = (*F*_o_^2^ + 2*F*_c_^2^)/3
*S* = 1.06	(Δ/σ)_max_ = 0.002
3300 reflections	Δρ_max_ = 0.39 e Å^−^^3^
240 parameters	Δρ_min_ = −0.19 e Å^−^^3^
0 restraints	

### Special details

**Table:**

Geometry. All esds (except the esd in the dihedral angle between two l.s. planes) are estimated using the full covariance matrix. The cell esds are taken into account individually in the estimation of esds in distances, angles and torsion angles; correlations between esds in cell parameters are only used when they are defined by crystal symmetry. An approximate (isotropic) treatment of cell esds is used for estimating esds involving l.s. planes.

### Fractional atomic coordinates and isotropic or equivalent isotropic displacement parameters (Å^2^)

**Table:**

	*x*	*y*	*z*	*U*_iso_*/*U*_eq_	Occ. (<1)
Sr1	0.74277 (2)	0.53023 (2)	0.46969 (2)	0.02003 (5)	
O1	1.03404 (12)	0.57713 (5)	0.53587 (9)	0.0276 (2)	
N1	0.71783 (17)	0.65620 (7)	0.33604 (12)	0.0315 (3)	
C1	1.14299 (18)	0.61161 (8)	0.60142 (13)	0.0248 (3)	
O2	1.29545 (13)	0.59817 (6)	0.60970 (11)	0.0350 (3)	
C2	1.0907 (2)	0.66959 (9)	0.67912 (16)	0.0333 (4)	
H2	0.967393	0.675584	0.660022	0.040*	
O3	0.44290 (13)	0.51634 (6)	0.36832 (9)	0.0277 (2)	
C3	1.1366 (3)	0.64916 (12)	0.80933 (18)	0.0564 (6)	
H3A	1.079880	0.605721	0.823650	0.085*	
H3B	1.102401	0.686203	0.859261	0.085*	
H3C	1.257147	0.642439	0.828695	0.085*	
O4	0.18685 (13)	0.48389 (7)	0.29816 (10)	0.0360 (3)	
C4	1.1738 (3)	0.73767 (10)	0.6536 (2)	0.0579 (6)	
H4A	1.294585	0.731263	0.665716	0.087*	
H4B	1.145448	0.773973	0.707215	0.087*	
H4C	1.134732	0.751626	0.571288	0.087*	
O5A	0.577 (3)	0.6350 (9)	0.5311 (11)	0.0265 (9)	0.381 (3)
H5A	0.609072	0.636661	0.604661	0.040*	0.381 (3)
C5	0.33403 (18)	0.49642 (8)	0.28442 (13)	0.0248 (3)	
O6A	0.882 (6)	0.530 (3)	0.273 (5)	0.0294 (17)	0.381 (3)
H6A	0.964326	0.504679	0.267529	0.044*	0.381 (3)
C6	0.3829 (2)	0.48362 (10)	0.16298 (15)	0.0350 (4)	
H6	0.502060	0.497040	0.166344	0.042*	
C7	0.2768 (3)	0.52822 (12)	0.06894 (18)	0.0542 (6)	
H7A	0.159415	0.515843	0.065063	0.081*	
H7B	0.310497	0.519856	−0.008377	0.081*	
H7C	0.292495	0.577568	0.089822	0.081*	
C8	0.3644 (3)	0.40680 (11)	0.13232 (17)	0.0484 (5)	
H8A	0.432387	0.379290	0.194290	0.073*	
H8B	0.401459	0.398322	0.056146	0.073*	
H8C	0.247570	0.393170	0.126731	0.073*	
C9A	0.5616 (6)	0.6538 (3)	0.2356 (5)	0.0484 (14)	0.381 (3)
H9AA	0.462861	0.641832	0.270231	0.073*	0.381 (3)
H9AB	0.578748	0.618626	0.176647	0.073*	0.381 (3)
H9AC	0.545747	0.699558	0.197278	0.073*	0.381 (3)
C10A	0.6919 (7)	0.7102 (2)	0.4143 (5)	0.0447 (13)	0.381 (3)
H10A	0.658994	0.752626	0.367158	0.054*	0.381 (3)
H10B	0.798802	0.720209	0.466068	0.054*	0.381 (3)
C11A	0.5675 (15)	0.6966 (8)	0.4890 (14)	0.0384 (12)	0.381 (3)
H11A	0.581608	0.730392	0.555314	0.046*	0.381 (3)
H11B	0.455628	0.703709	0.442146	0.046*	0.381 (3)
C12A	0.8593 (6)	0.6604 (2)	0.2824 (4)	0.0372 (12)	0.381 (3)
H12A	0.957084	0.667164	0.345023	0.045*	0.381 (3)
H12B	0.849687	0.702121	0.230877	0.045*	0.381 (3)
C13A	0.893 (4)	0.5941 (18)	0.205 (3)	0.0431 (8)	0.381 (3)
H13A	0.809510	0.592742	0.131210	0.052*	0.381 (3)
H13B	1.004460	0.597745	0.181722	0.052*	0.381 (3)
O6	0.866 (3)	0.5342 (16)	0.268 (3)	0.0294 (17)	0.619 (3)
H6B	0.960128	0.517184	0.294366	0.044*	0.619 (3)
O5	0.5875 (15)	0.6406 (5)	0.5558 (6)	0.0265 (9)	0.619 (3)
H5	0.505903	0.619209	0.574990	0.040*	0.619 (3)
C9	0.8519 (4)	0.70964 (15)	0.3830 (3)	0.0411 (7)	0.619 (3)
H9A	0.961638	0.690803	0.376032	0.062*	0.619 (3)
H9B	0.846963	0.719638	0.466490	0.062*	0.619 (3)
H9C	0.832910	0.752678	0.336686	0.062*	0.619 (3)
C11	0.5250 (7)	0.6997 (5)	0.4694 (8)	0.0384 (12)	0.619 (3)
H11C	0.403861	0.705598	0.467052	0.046*	0.619 (3)
H11D	0.580042	0.743736	0.498683	0.046*	0.619 (3)
C12	0.7389 (4)	0.63928 (16)	0.2165 (2)	0.0404 (8)	0.619 (3)
H12C	0.747000	0.683090	0.172108	0.048*	0.619 (3)
H12D	0.639044	0.613961	0.177495	0.048*	0.619 (3)
C13	0.887 (2)	0.5965 (11)	0.2096 (17)	0.0431 (8)	0.619 (3)
H13C	0.896781	0.587366	0.125768	0.052*	0.619 (3)
H13D	0.988442	0.620832	0.247982	0.052*	0.619 (3)
C10	0.5591 (3)	0.68558 (14)	0.3441 (3)	0.0372 (7)	0.619 (3)
H10C	0.472220	0.653633	0.304369	0.045*	0.619 (3)
H10D	0.548179	0.730028	0.299556	0.045*	0.619 (3)

### Atomic displacement parameters (Å^2^)

**Table:**

	*U*^11^	*U*^22^	*U*^33^	*U*^12^	*U*^13^	*U*^23^
Sr1	0.01411 (8)	0.02466 (8)	0.02156 (8)	−0.00039 (4)	0.00361 (5)	−0.00108 (5)
O1	0.0218 (5)	0.0283 (5)	0.0319 (6)	−0.0029 (4)	0.0016 (4)	−0.0046 (4)
N1	0.0321 (8)	0.0296 (7)	0.0338 (7)	0.0040 (5)	0.0082 (6)	0.0048 (6)
C1	0.0201 (7)	0.0262 (7)	0.0287 (7)	−0.0014 (5)	0.0059 (5)	−0.0009 (6)
O2	0.0176 (6)	0.0348 (6)	0.0537 (7)	−0.0017 (4)	0.0092 (5)	−0.0128 (5)
C2	0.0233 (8)	0.0343 (8)	0.0418 (9)	0.0036 (6)	0.0040 (6)	−0.0114 (7)
O3	0.0203 (5)	0.0360 (6)	0.0259 (5)	−0.0021 (4)	0.0010 (4)	−0.0006 (4)
C3	0.0662 (14)	0.0652 (14)	0.0378 (11)	0.0156 (11)	0.0084 (9)	−0.0165 (10)
O4	0.0177 (6)	0.0627 (8)	0.0276 (6)	−0.0007 (5)	0.0043 (4)	0.0012 (5)
C4	0.0619 (14)	0.0340 (10)	0.0818 (16)	−0.0043 (9)	0.0239 (11)	−0.0198 (10)
O5A	0.0273 (17)	0.0322 (16)	0.021 (3)	−0.0033 (11)	0.008 (3)	−0.0085 (19)
C5	0.0200 (8)	0.0308 (8)	0.0235 (7)	0.0022 (6)	0.0031 (5)	0.0009 (6)
O6A	0.023 (4)	0.035 (3)	0.031 (2)	0.007 (3)	0.007 (3)	0.0037 (18)
C6	0.0265 (9)	0.0539 (10)	0.0260 (8)	−0.0021 (7)	0.0088 (6)	−0.0036 (7)
C7	0.0684 (15)	0.0683 (14)	0.0276 (9)	0.0088 (11)	0.0131 (9)	0.0082 (9)
C8	0.0536 (12)	0.0571 (12)	0.0346 (10)	0.0073 (9)	0.0073 (8)	−0.0139 (9)
C9A	0.037 (3)	0.052 (3)	0.052 (3)	−0.001 (2)	−0.005 (2)	0.017 (2)
C10A	0.057 (3)	0.026 (2)	0.056 (3)	−0.003 (2)	0.022 (2)	−0.002 (2)
C11A	0.030 (3)	0.0315 (12)	0.056 (3)	0.017 (2)	0.015 (3)	0.0070 (18)
C12A	0.039 (3)	0.034 (2)	0.042 (3)	0.0017 (18)	0.015 (2)	0.015 (2)
C13A	0.0521 (15)	0.0471 (17)	0.0352 (18)	0.0102 (11)	0.0227 (11)	0.0109 (12)
O6	0.023 (4)	0.035 (3)	0.031 (2)	0.007 (3)	0.007 (3)	0.0037 (18)
O5	0.0273 (17)	0.0322 (16)	0.021 (3)	−0.0033 (11)	0.008 (3)	−0.0085 (19)
C9	0.0362 (16)	0.0362 (15)	0.0495 (18)	−0.0081 (12)	0.0024 (12)	0.0055 (13)
C11	0.030 (3)	0.0315 (12)	0.056 (3)	0.017 (2)	0.015 (3)	0.0070 (18)
C12	0.051 (2)	0.0420 (16)	0.0275 (14)	0.0089 (13)	0.0056 (12)	0.0089 (12)
C13	0.0521 (15)	0.0471 (17)	0.0352 (18)	0.0102 (11)	0.0227 (11)	0.0109 (12)
C10	0.0311 (15)	0.0330 (14)	0.0478 (17)	0.0068 (11)	0.0076 (12)	0.0106 (12)

### Geometric parameters (Å, º)

**Table:**

Sr1—O1^i^	2.7563 (10)	C6—H6	1.0000
Sr1—O3^ii^	2.7244 (11)	C6—C7	1.528 (3)
Sr1—O1	2.5377 (10)	C6—C8	1.517 (3)
Sr1—O3	2.5444 (10)	C7—H7A	0.9800
Sr1—O2^i^	2.6270 (11)	C7—H7B	0.9800
Sr1—O4^ii^	2.6364 (11)	C7—H7C	0.9800
Sr1—O5	2.731 (11)	C8—H8A	0.9800
Sr1—O6	2.66 (3)	C8—H8B	0.9800
Sr1—N1	2.8495 (13)	C8—H8C	0.9800
Sr1—Sr1^i^	4.2981 (3)	C9A—H9AA	0.9800
Sr1—Sr1^ii^	4.2868 (3)	C9A—H9AB	0.9800
Sr1—O5A	2.58 (2)	C9A—H9AC	0.9800
Sr1—C5^ii^	3.0199 (15)	C10A—H10A	0.9900
Sr1—O6A	2.68 (6)	C10A—H10B	0.9900
O1—C1	1.2541 (18)	C10A—C11A	1.450 (16)
N1—C9A	1.571 (5)	C11A—H11A	0.9900
N1—C10A	1.407 (5)	C11A—H11B	0.9900
N1—C12A	1.393 (5)	C12A—H12A	0.9900
N1—C9	1.531 (3)	C12A—H12B	0.9900
N1—C12	1.441 (3)	C12A—C13A	1.60 (3)
N1—C10	1.428 (3)	C13A—H13A	0.9900
C1—O2	1.2578 (19)	C13A—H13B	0.9900
C1—C2	1.527 (2)	O6—H6B	0.8400
C2—H2	1.0000	O6—C13	1.39 (4)
C2—C3	1.526 (3)	O5—H5	0.8400
C2—C4	1.521 (3)	O5—C11	1.537 (12)
O3—C5	1.2563 (18)	C9—H9A	0.9800
C3—H3A	0.9800	C9—H9B	0.9800
C3—H3B	0.9800	C9—H9C	0.9800
C3—H3C	0.9800	C11—H11C	0.9900
O4—C5	1.2581 (19)	C11—H11D	0.9900
C4—H4A	0.9800	C11—C10	1.526 (10)
C4—H4B	0.9800	C12—H12C	0.9900
C4—H4C	0.9800	C12—H12D	0.9900
O5A—H5A	0.8400	C12—C13	1.471 (18)
O5A—C11A	1.27 (2)	C13—H13C	0.9900
C5—C6	1.524 (2)	C13—H13D	0.9900
O6A—H6A	0.8400	C10—H10C	0.9900
O6A—C13A	1.46 (6)	C10—H10D	0.9900
			
O1—Sr1—O1^i^	71.51 (4)	H4A—C4—H4C	109.5
O1—Sr1—N1	80.86 (4)	H4B—C4—H4C	109.5
O1^i^—Sr1—N1	127.74 (4)	Sr1—O5A—H5A	102.1
O1—Sr1—O2^i^	119.26 (3)	C11A—O5A—Sr1	128.5 (14)
O1—Sr1—O3	163.08 (4)	C11A—O5A—H5A	109.5
O1—Sr1—O3^ii^	120.63 (3)	O3—C5—Sr1^ii^	64.42 (8)
O1—Sr1—O4^ii^	72.27 (3)	O3—C5—O4	122.22 (14)
O1—Sr1—O5A	98.5 (4)	O3—C5—C6	119.20 (14)
O1^i^—Sr1—C5^ii^	97.57 (4)	O4—C5—Sr1^ii^	60.41 (8)
O1—Sr1—C5^ii^	96.16 (4)	O4—C5—C6	118.51 (13)
O1—Sr1—O6A	75.4 (11)	C6—C5—Sr1^ii^	160.72 (11)
O1—Sr1—O6	77.3 (6)	Sr1—O6A—H6A	120.7
O1—Sr1—O5	94.8 (2)	C13A—O6A—Sr1	121 (3)
N1—Sr1—C5^ii^	129.42 (4)	C13A—O6A—H6A	109.5
O2^i^—Sr1—O1^i^	48.14 (3)	C5—C6—H6	108.6
O2^i^—Sr1—N1	128.06 (4)	C5—C6—C7	110.42 (15)
O2^i^—Sr1—O3^ii^	83.06 (4)	C7—C6—H6	108.6
O2^i^—Sr1—O4^ii^	104.22 (4)	C8—C6—C5	109.65 (15)
O2^i^—Sr1—C5^ii^	97.57 (4)	C8—C6—H6	108.6
O2^i^—Sr1—O6A	75.6 (12)	C8—C6—C7	110.99 (16)
O2^i^—Sr1—O6	76.6 (7)	C6—C7—H7A	109.5
O2^i^—Sr1—O5	144.7 (2)	C6—C7—H7B	109.5
O3—Sr1—O1^i^	120.02 (3)	C6—C7—H7C	109.5
O3^ii^—Sr1—O1^i^	102.22 (3)	H7A—C7—H7B	109.5
O3—Sr1—N1	82.22 (4)	H7A—C7—H7C	109.5
O3^ii^—Sr1—N1	130.04 (4)	H7B—C7—H7C	109.5
O3—Sr1—O2^i^	72.11 (3)	C6—C8—H8A	109.5
O3—Sr1—O3^ii^	71.14 (4)	C6—C8—H8B	109.5
O3—Sr1—O4^ii^	118.97 (3)	C6—C8—H8C	109.5
O3—Sr1—O5A	72.5 (4)	H8A—C8—H8B	109.5
O3—Sr1—C5^ii^	94.46 (4)	H8A—C8—H8C	109.5
O3^ii^—Sr1—C5^ii^	24.58 (4)	H8B—C8—H8C	109.5
O3—Sr1—O6A	97.1 (10)	N1—C9A—H9AA	109.5
O3—Sr1—O6	94.5 (6)	N1—C9A—H9AB	109.5
O3^ii^—Sr1—O5	70.7 (2)	N1—C9A—H9AC	109.5
O3—Sr1—O5	77.2 (2)	H9AA—C9A—H9AB	109.5
O4^ii^—Sr1—O1^i^	84.80 (3)	H9AA—C9A—H9AC	109.5
O4^ii^—Sr1—N1	127.72 (4)	H9AB—C9A—H9AC	109.5
O4^ii^—Sr1—O3^ii^	48.46 (3)	N1—C10A—H10A	108.4
O4^ii^—Sr1—C5^ii^	24.52 (4)	N1—C10A—H10B	108.4
O4^ii^—Sr1—O6A	142.4 (9)	N1—C10A—C11A	115.7 (8)
O4^ii^—Sr1—O6	145.4 (6)	H10A—C10A—H10B	107.4
O4^ii^—Sr1—O5	75.82 (16)	C11A—C10A—H10A	108.4
O5A—Sr1—N1	59.3 (4)	C11A—C10A—H10B	108.4
O5A—Sr1—C5^ii^	71.5 (4)	O5A—C11A—C10A	112.5 (14)
O5A—Sr1—O6A	122.7 (11)	O5A—C11A—H11A	109.1
O6A—Sr1—N1	63.5 (10)	O5A—C11A—H11B	109.1
O6A—Sr1—C5^ii^	164.0 (9)	C10A—C11A—H11A	109.1
O6—Sr1—O1^i^	70.0 (6)	C10A—C11A—H11B	109.1
O6—Sr1—N1	61.0 (6)	H11A—C11A—H11B	107.8
O6—Sr1—O3^ii^	158.1 (7)	N1—C12A—H12A	108.5
O6—Sr1—C5^ii^	167.2 (5)	N1—C12A—H12B	108.5
O6—Sr1—O5	123.5 (7)	N1—C12A—C13A	115.2 (12)
O5—Sr1—O1^i^	159.08 (16)	H12A—C12A—H12B	107.5
O5—Sr1—N1	62.5 (2)	C13A—C12A—H12A	108.5
O5—Sr1—C5^ii^	67.5 (2)	C13A—C12A—H12B	108.5
Sr1—O1—Sr1^i^	108.50 (4)	O6A—C13A—C12A	110 (3)
Sr1—O1—Sr1^ii^	108.84 (5)	O6A—C13A—H13A	109.7
C1—O1—Sr1	154.61 (10)	O6A—C13A—H13B	109.7
C1—O1—Sr1^i^	90.10 (9)	C12A—C13A—H13A	109.7
C9A—N1—Sr1	110.3 (2)	C12A—C13A—H13B	109.7
C10A—N1—Sr1	106.8 (2)	H13A—C13A—H13B	108.2
C10A—N1—C9A	107.4 (3)	Sr1—O6—H6B	97.5
C12A—N1—Sr1	106.99 (19)	C13—O6—Sr1	122 (2)
C12A—N1—C9A	108.1 (3)	C13—O6—H6B	109.5
C12A—N1—C10A	117.2 (3)	Sr1—O5—H5	98.5
C9—N1—Sr1	113.27 (13)	C11—O5—Sr1	117.7 (5)
C12—N1—Sr1	107.93 (14)	C11—O5—H5	109.5
C12—N1—C9	107.2 (2)	N1—C9—H9A	109.5
C10—N1—Sr1	106.71 (13)	N1—C9—H9B	109.5
C10—N1—C9	108.39 (19)	N1—C9—H9C	109.5
C10—N1—C12	113.45 (19)	H9A—C9—H9B	109.5
O1—C1—Sr1^i^	65.45 (8)	H9A—C9—H9C	109.5
O1—C1—O2	122.18 (14)	H9B—C9—H9C	109.5
O1—C1—C2	119.64 (13)	O5—C11—H11C	109.1
O2—C1—Sr1^i^	59.55 (8)	O5—C11—H11D	109.1
O2—C1—C2	118.10 (13)	H11C—C11—H11D	107.9
C2—C1—Sr1^i^	159.64 (11)	C10—C11—O5	112.4 (6)
C1—O2—Sr1^i^	96.07 (9)	C10—C11—H11C	109.1
C1—C2—H2	108.8	C10—C11—H11D	109.1
C3—C2—C1	109.41 (14)	N1—C12—H12C	108.8
C3—C2—H2	108.8	N1—C12—H12D	108.8
C4—C2—C1	109.84 (15)	N1—C12—C13	113.6 (8)
C4—C2—H2	108.8	H12C—C12—H12D	107.7
C4—C2—C3	111.17 (17)	C13—C12—H12C	108.8
Sr1—O3—Sr1^ii^	108.86 (4)	C13—C12—H12D	108.8
C5—O3—Sr1^ii^	91.00 (9)	O6—C13—C12	106.9 (17)
C5—O3—Sr1	152.58 (10)	O6—C13—H13C	110.3
C2—C3—H3A	109.5	O6—C13—H13D	110.3
C2—C3—H3B	109.5	C12—C13—H13C	110.3
C2—C3—H3C	109.5	C12—C13—H13D	110.3
H3A—C3—H3B	109.5	H13C—C13—H13D	108.6
H3A—C3—H3C	109.5	N1—C10—C11	115.8 (3)
H3B—C3—H3C	109.5	N1—C10—H10C	108.3
C5—O4—Sr1^ii^	95.07 (9)	N1—C10—H10D	108.3
C2—C4—H4A	109.5	C11—C10—H10C	108.3
C2—C4—H4B	109.5	C11—C10—H10D	108.3
C2—C4—H4C	109.5	H10C—C10—H10D	107.4
H4A—C4—H4B	109.5		
			
Sr1—O1—C1—Sr1^i^	138.0 (2)	Sr1—O5—C11—C10	1.0 (9)
Sr1^i^—O1—C1—O2	18.98 (15)	O1—C1—O2—Sr1^i^	−20.07 (16)
Sr1—O1—C1—O2	157.02 (16)	O1—C1—C2—C3	113.07 (18)
Sr1^i^—O1—C1—C2	−157.82 (13)	O1—C1—C2—C4	−124.64 (17)
Sr1—O1—C1—C2	−19.8 (3)	N1—C10A—C11A—O5A	42.3 (14)
Sr1—N1—C10A—C11A	−46.2 (7)	N1—C12A—C13A—O6A	−49 (3)
Sr1—N1—C12A—C13A	54.3 (13)	N1—C12—C13—O6	61.3 (18)
Sr1—N1—C12—C13	−50.4 (9)	O2—C1—C2—C3	−63.9 (2)
Sr1—N1—C10—C11	57.0 (5)	O2—C1—C2—C4	58.4 (2)
Sr1^i^—C1—C2—C3	13.9 (4)	C2—C1—O2—Sr1^i^	156.78 (12)
Sr1^i^—C1—C2—C4	136.2 (3)	O3—C5—C6—C7	−123.02 (18)
Sr1—O3—C5—Sr1^ii^	137.4 (2)	O3—C5—C6—C8	114.37 (17)
Sr1^ii^—O3—C5—O4	18.43 (16)	O4—C5—C6—C7	59.8 (2)
Sr1—O3—C5—O4	155.79 (15)	O4—C5—C6—C8	−62.8 (2)
Sr1^ii^—O3—C5—C6	−158.64 (13)	C9A—N1—C10A—C11A	72.0 (7)
Sr1—O3—C5—C6	−21.3 (3)	C9A—N1—C12A—C13A	−64.4 (13)
Sr1^ii^—O4—C5—O3	−19.14 (16)	C10A—N1—C12A—C13A	174.1 (13)
Sr1^ii^—O4—C5—C6	157.94 (13)	C12A—N1—C10A—C11A	−166.1 (6)
Sr1—O5A—C11A—C10A	−12.8 (19)	O5—C11—C10—N1	−41.3 (8)
Sr1^ii^—C5—C6—C7	141.2 (3)	C9—N1—C12—C13	72.0 (9)
Sr1^ii^—C5—C6—C8	18.6 (4)	C9—N1—C10—C11	−65.3 (5)
Sr1—O6A—C13A—C12A	15 (4)	C12—N1—C10—C11	175.7 (5)
Sr1—O6—C13—C12	−41 (2)	C10—N1—C12—C13	−168.4 (9)

Symmetry codes: (i) −*x*+2, −*y*+1, −*z*+1; (ii) −*x*+1, −*y*+1, −*z*+1.

### Hydrogen-bond geometry (Å, º)

**Table:**

*D*—H···*A*	*D*—H	H···*A*	*D*···*A*	*D*—H···*A*
O6*A*—H6*A*···O4^iii^	0.84	1.83	2.61 (5)	153
O6—H6*B*···O4^iii^	0.84	1.95	2.75 (3)	160
O5—H5···O2^iv^	0.84	1.87	2.680 (12)	163

Symmetry codes: (iii) *x*+1, *y*, *z*; (iv) *x*−1, *y*, *z*.

### Selected bond lengths (Å) and angles (°).

**Table:**

Sr1 – O1^i^	2.7568 (13)	Sr1 – O6	2.655 (4)
Sr1 – O3^ii^	2.7251 (14)	Sr1 – N1	2.8516 (16)
Sr1 – O1	2.5370 (12)	Sr1 ··· Sr1^i^	4.2981 (3)
Sr1 – O3	2.5441 (13)	Sr1 ··· Sr1^ii^	4.2868 (3)
Sr1 – O2^i^	2.6273 (14)	Sr1 – O1 – Sr1^i^	108.50 (4)
Sr1 – O4^ii^	2.6367 (14)	Sr1 – O1 – Sr1^ii^	108.84 (5)
Sr1 – O5	2.678 (4)		

Symmetry codes: (i) 2 – x, 1 – y, 1 – z; (ii) 1 – x, 1 – y, 1 – z.
