# Supplementary material for: Synthesis and crystal structure of a one-dimensional chain-like strontium(II) coordination polymer built of N-methyldi­ethano­lamine and isobutyrate ligands
Source: Acta Crystallogr E Crystallogr Commun. 2021 Jun 11;77(Pt 7):703–7. doi: 10.1107/S2056989021005594 (PMC8382055; doi:10.1107/S2056989021005594)
Supplement: Supplementary file 3 [file e-77-00703-sup4.zip › Acta Cryst E revised.pdf]

Synthesis and crystal structure of a one-dimensional chain-like strontium(II) coordination polymer built of *N*-methyldiethanolamine and isobutyrate ligands

Maximilian Seiß, Sebastian Schmitz, Martin Börner and Kirill Yu. Monakhov*

Leibniz Institute of Surface Engineering (IOM), Permoserstr. 15, 04318 Leipzig, Germany

Correspondence email: kirill.monakhov@iom-leipzig.de

Abstract

The one-dimensional coordination polymer (**I**) $[Sr(ib)_2(H_2mda)]_n$ (Hib = isobutyric acid and H_2mda = *N*-methyldiethanolamine), namely, *catena*-poly[[(*N*-methyldiethanolamine-κ^3N,O,O')-strontium(II)]-(di-μ_2-isobutyrate-$\kappa^3O,O':O$)-(di-μ_2-isobutyrate-$\kappa^3O:O,O'$)], was prepared by the one-pot aerobic reaction of $[Zr_6O_4(OH)_4(ib)_{12}(H_2O)]\cdot 3Hib$ with $Sr(NO_3)_2$ and H_2mda in the presence of $MnCl_2$ and Et_3N in acetonitrile. The use of $MnCl_2$ is key to the isolation of **I** as high-quality colorless crystals in good yield. The molecular solid-state structure of **I** was determined by single-crystal X-ray diffraction. **I** crystallizes in the monoclinic space group $P2_1/c$ and shows a one-dimensional polymeric chain structure. Each monomeric unit of this coordination polymer consists of a central Sr(II) ion in the NO_8 coordination environment of two deprotonated ib^- ligands and one fully protonated H_2mda ligand. **I** shows thermal stability up to 130 °C in air.

1. Chemical context

Simple metal isobutyrate salts such as $TM(ib)_2$ (*e.g.* TM = Mn, Co and Ni; Hib = isobutyric acid) and AM(ib) (*e.g.* AM = Na and K) are known to act as precursor materials for the synthesis of a wide variety of polynuclear coordination complexes, *e.g.* $[Mn^{II}_4Mn^{III}_2(ib)_8(Hbda)_2(bda)_2]$ (H_2bda = *N*-butyldiethanolamine), $[Mn^{II}_4Co^{III}_2(ib)_8(Hmda)_2(mda)_2]$ (Malaestean *et al.*, 2010), $[Co^{II}_3Co^{III}_2(Hbda)_2(bda)_2(ib)_6]\cdot 2MeCN$ and $[Ni^{II}_4(Hbda)_3(ib)_5(MeCN)]$ (Schmitz *et al.*, 2016), $[Gd^{III}_4M^{II}_8(OH)_8(Lig)_8(ib)_8](ClO_4)_4$ (M = Zn(II) or Cu(II), HLig = 2-(hydroxymethyl)pyridine) (Hooper *et al.*, 2012) and $[Cr_3O(ib)_6(H_2O)_3](NO_3)$ (Parsons *et al.*, 2000). The formation of these polynuclear homo- and heterometallic complexes has been enabled due to the introduction of flexible amino alcohol ligands into the reaction mixtures (Schmitz *et al.*, 2016; Malaestean *et al.*, 2010). We here describe the first example of a coordination polymer composed of monomeric Sr(II) units that are supported by both isobutyrate- and amino alcohol ligands (Scheme 1). This makes the synthesized compound $[Sr(ib)_2(H_2mda)]_n$ (**I**) an appealing precursor for reactions with transition metal and lanthanide complexes. Also **I** can find application in solvothermal reactions as it is described *e.g.* for the transformation of $[Co^{II}_3Co^{III}_2(Hbda)_2(bda)_2(ib)_6]$ to $[Co^{II}_{10}(OH)_2(bda)_6(ib)_6]$ (Schmitz *et al.*, 2018). Herein compound **I** was isolated as colorless crystals from an aerobic reaction, characterized by infrared (IR) spectroscopy, thermogravimetric analysis (TGA) and single-crystal X-ray diffraction. **I** represents a rare class of alkaline earth metal-isobutyrate complexes with one-dimensional (1D) polymeric structure (*cf.* $\{[Mg(ib)_2(H_2O)_3]\cdot H_2O\}_n$ (Malaestean *et al.*, 2013)). Remarkably, $MnCl_2$ in the synthesis of **I** is crucial for the formation of high-quality single crystals (in 36% yield) suitable for X-ray diffraction. When the reaction is carried out without $MnCl_2$, poor quality crystalline material is formed in lower yield within several days. The IR spectrum of **I** is characterized by the asymmetric O–C–O vibration bands at 1556 cm^{-1} and the symmetric O–C–O ones in the range of 1366–1426 cm^{-1}. For the syntheses of homometallic coordination complexes it is known to use an additional metal salt, which yields a heterometallic reaction mixture, from which the homometallic complex can be obtained as solid product selectively (Ako *et al.*, 2007; Liu *et al.*, 2018). In 2007 Ako *et al.* described two heptanuclear iron(III) complexes $[Fe^{III}_7O_3(bda)_3(piv)_9(H_2O)_3]$ and $[Fe^{III}_7O_3(phda)_3(piv)_9(H_2O)_3]$ (H_2bda = *N*-butyl-

diethanolamine, H_2phda = *N*-phenyldiethanolamine and Hpiv = pivalic acid), which were obtained by the reaction of [Fe_3O(piv)$_6$]piv, nickel(II) acetate tetrahydrate ($Ni(OAc)_2·4H_2O$) and H_2bda or H_2phda in a molar ratio of 1:1:2 using MeCN as solvent (Ako *et al.*, 2007). Although $Ni(OAc)_2·4H_2O$ was used in an equimolar ratio with the iron(III) precursor, nickel did not incorporate into the final product. Similar to this Liu *et al.* synthesized an hexanuclear [Zn_6(Lig)$_6$($OOCH$)$_6$] complex (HLig = 4'-(4-carboxyphenyl)-2,2':6',2''-terpyridine) by the reaction of $Zn(OAc)_2$ with HLig in the presence of praseodym(III) nitrate hexahydrate ($Pr(NO_3)_3·6H_2O$) using a 2:2:1 molar ratio. The reaction was performed solvothermal in DMF and praseodym did not incorporate into the final [Zn_6(Lig)$_6$($OOCH$)$_6$] complex, which was isolated as pure product by filtration (Liu *et al.*, 2018). Here [Sr(ib)$_2$(H_2mda)]$_n$ was as well isolated as pure product by filtration, which indicates that the additional metal salts (here $MnCl_2$) remain in the mother liquor.

2. Structural commentary

The crystal structure consists of a Sr(II) monomer unit (Figure 1) extended along the *a* axis. The asymmetric unit contains one central Sr(II) ion, which is coordinated by a disordered, tridentate and fully protonated H_2mda and two deprotonated isobutyrate ligands. In other words, Sr(II) is nine-coordinated by six O atoms (O1, O3, O1^{i}, O3^{ii}, O2^{i}, and O4^{ii}; see Table 1 for geometric parameters and symmetry codes) from four different carboxylate groups, two O atoms (O5 and O6 or O5A and O6A) and one N atom (N1) from the *N*-methyldiethanolamine ligand. The resulting coordination environment of strontium center is NO_8. The polyhedral shape of Sr was evaluated using the SHAPE software version 2.1 (Llunell *et al.*, 2013) and can be described as an in-between a distorted spherical capped square anti-prism and a distorted spherical tricapped trigonal prism (Figure 2). The values of the deviation from the ideal geometry are listed in Table 2. The Sr–O_{ib} bond lengths of the bridging O atoms are in between 2.5377 (10) Å and 2.7563 (10) Å, whereas the non-bridging Sr–O_{ib} bond lengths range from 2.6270 (11) Å to 2.6364 (11) Å. The non-bonding Sr···Sr distances are 4.2869 (3) Å and 4.2982 (3) Å with Sr–O–Sr angles of 108.50 (4)° and 108.86 (5)°. The Sr–O_{H2mda} bond lengths are between 2.582 (20) Å and 2.731 (11) Å, and Sr–N bond lengths are 2.8495 (13) Å.

3. Supramolecular features

The crystal packing reveals the existence of 1D polymeric zigzag chains lying along the *a* axis (Figures 3, 4), where monomeric Sr(II) units are interlinked by one O atom of each isobutyrate ligand, which are all coordinated in a chelating, bridging μ_2-η^2:η^1 mode. The H_2mda ligands coordinate in the chelating μ_1-η^1: η^1: η^1 mode to the Sr centers of **I**. The edge-sharing $SrNO_8$ polyhedra are linked by the isobutyrate O1 and O1^{ii} atoms on the one side and O3 and O3^{i} atoms on the other side. Intramolecular hydrogen bonding is present along the chains *via* O5–H5···O2, O6–H6···O4 and O6A–H6A···O4 contacts (Figure 3, Table 3).

4. Database survey

A search of the Cambridge Structural Database (CSD, version 5.42, update November 2020; Groom *et al.*, 2016) resulted in 34 hits for metal complexes ligated by isobutyrate and *N*-alkyldiethanolamine. To the best of our knowledge, there are no alkaline earth complexes as well as coordination polymers incorporating both ligands. There are four polymeric structures solely containig group two elements and isobutyrate anions. The magnesium complex *catena*-poly[[triaqua(isobutyrato)-κ*O*)magnesium]-*μ*-isobutyrato-κ^2*O:O'*] monohydrate, refcode VIQTOG (Malaestean *et al.*, 2013), *catena*-poly[[*μ*-aqua-diaqua(*μ*$_3$-2-methylpropanoato-κ^4 *O:O,O':O'*)calcium] 2-methylpropanoate dihydrate], refcode JUWMEW (Samolová *et al.*, 2020), as well as the isostructural strontium complex, refcode JUWMIA (Samolová *et al.*, 2020) and the mixed calcium/strontium complex *catena*-poly[[*μ*-aqua-diaqua(*μ*$_3$-2-methylpropanoato-κ^4*O:O,O':O'*)calcium/strontium] 2-methylpropanoate dihydrate], refcode JUWMOG (Samolová *et al.*, 2020).

5. Synthesis and crystallization

The one-pot reaction of freshly prepared hexanuclear zirconium complex $[Zr_6O_4(OH)_4(ib)_{12}(H_2O)]\cdot3Hib$ (Kogler *et al.*, 2004, abbreviated as $\{Zr_6\}$) with strontium(II) nitrate and manganese(II) chloride in a 1.0:2.2:2.2, molar ratio was performed in acetonitrile under aerobic conditions, involving 11.1 eq. of *N*-methyldiethanolamine as a co-ligand and 4.0 eq. of triethylamine as a base (see Figure 5). The polymeric coordination complex $[Sr(ib)_2(H_2mda)]_n$ (**I**) was isolated as colorless crystals. By-products could not be identified.

The TGA curve (Figure 6) shows that the thermal decomposition of **I** occurs between 130 °C and 440 °C with a mass loss of $C_{12}H_{27}NO_3$ per monomer unit (Δm_{total} = 60.00% *vs*. $\Delta m_{calcd.}$ = 61.25%), and it yields $SrCO_3$. Overall, the thermal stability of **I** up to 130°C in air is similar to that determined for isobutyrate diethanolamine complexes of cobalt (140 °C) and nickel (130 °C) (Schmitz *et al.*, 2016).

6. Refinement

Crystal data, data collection and structure refinement details are summarized in Table 4. The structure was solved using dual methods and refined by full matrix least squares minimization on F^2. The coordinates of all non-hydrogen atoms were refined with anisotropic thermal parameters. All H atoms were placed in geometrically idealized positions and refined using a rigid model and included as riding atoms, with methyl C–H = 0.98 Å, methylene C–H = 0.99 Å, methine C–H = 1.00 Å and O–H = 0.84 Å. Isotropic displacement parameters were set to $U_{iso}(H) = 1.2U_{eq}$ for the parent atom (1.5 for methyl and hydroxy groups). The hydroxy groups and the idealized methyl group were refined as rotating. Atoms C9, C10, C11, C12, C13, O5 and O6 of the H_2mda ligand were refined as disordered over two sets of sites with site occupancies of 0.619 (3) and 0.381 (3). Due to a short distance between the disordered atoms C11, C13, O5, O6 and their corresponding counterpart EADP constrains were applied to equalize the thermal ellipsoids of the atom pairs.

Table 1
Experimental details

Crystal data	
Chemical formula	$C_{13}H_{27}NO_6Sr$
M_r	380.97
Crystal system, space group	Monoclinic, $P2_1/c$
Temperature (K)	180
a, b, c (Å)	8.1516 (2), 19.1921 (6), 11.4288 (3)
β (°)	99.295 (2)
V (Å^3)	1764.52 (8)
Z	4
Radiation type	Cu $K\alpha$
μ (mm^{-1})	4.46
Crystal size (mm)	0.28 × 0.21 × 0.13
Data collection	
Diffractometer	STOE STADIVARI
Absorption correction	Multi-scan<br>STOE LANA, absorption correction by scaling of reflection intensities. J. Koziskova, F. Hahn, J. Richter, J. Kozisek, "Comparison of different absorption corrections on the model structure of tetrakis(μ$_2$-acetato)-diaqua-di-copper(II)", Acta Chimica Slovaca, vol. 9, no. 2, 2016, pp. 136 - 140.
T_{min}, T_{max}	0.178, 0.458

No. of measured, independent and observed reflections	15496, 3300, 3009 {$I > 2\sigma(I)$}
R_{int}	0.014
$(\sin \theta/\lambda)_{max}$ (Å^{-1})	0.611
Refinement	
$R[F^2 > 2\sigma(F^2)]$, $wR(F^2)$, S	0.018, 0.046, 1.06
No. of reflections	3300
No. of parameters	240
H-atom treatment	H-atom parameters constrained
$\Delta\rho_{max}$, $\Delta\rho_{min}$ (e Å^{-3})	0.39, −0.19

Computer programs: *X-AREA* Pilatus3_SV 1.31.154.0 (STOE, 2019), *X-AREA* Recipe 1.34.0.0 (STOE, 2019), *X-AREA* Integrate 1.77.0.0 (STOE, 2019) *X-AREA* LANA 1.77.1.0 (STOE, 2019), olex2.solve 1.3 (Bourhis *et al.*, 2015), *SHELXL* 2018/3 (Sheldrick, 2015), Olex2 1.3 (Dolomanov *et al.*, 2009), STOE & Cie GmbH, *X-AREA*, software package for collecting single-crystal or multi-domain crystal data on STOE area-detector diffractometers, for image processing, for the correction and scaling of reflection intensities and for outlier rejection, version 1.88, Darmstadt 2019.

Acknowledgements

The authors are grateful to Professor H. Krautscheid (Leipzig University) for access to the single-crystal X-ray diffraction equipment.

References

Ako, A. M., Waldmann, O., Mereacre, V., Klöwer, F., Hewitt, I. J., Anson, C. E., Güdel, H. U. & &PowellA, K. (2007). *Inorg. Chem.* **46**, 756–766.

Bourhis, L. J., Dolomanov, O. V., Gildea, R. J., Howard, J. A. K. & Puschmann, H. (2015). *Acta Cryst.* A**71**, 59–75.

Dolomanov, O. V., Bourhis, L. J., Gildea, R. J., Howard, J. A. K. & Puschmann, H. (2009). *J. Appl. Cryst.* **42**, 339–341.

Groom, C. R., Bruno, I. J., Lightfoot, M. P. & Ward, S. C. (2016). *Acta Cryst*. B**72**, 171–179.

Hooper, T. N., Schnack, J., Piligkos, S., Evangelisti, M. & Brechin, E. K. (2012). *Angew. Chem. Int. Ed.* **51**, 4633–4636.

Kogler, F. R., Jupa, M., Puchberger, M. & Schubert, U. (2004). *J. Mater. Chem.* **14**, 3133–3138.

Liu, X., Gao, P. & Hu, M. (2018). *Polyhedron* **144**,119–124.

Llunell, M., Casanova, D., Cirera, A., Alemany, A., Alvarez, S., *SHAPE* version 2.1. (2013). University of Barcelona, Barcelona, Spain.

Malaestean, I. L., Speldrich, M., Ellern, A., Baca, S. G. & Kögerler, P. (2010). *Polyhedron*, **29**, 1990–1997.

Malaestean, I. L., Schmitz, S., Ellern, A. & Kögerler, P. (2013). *Acta Cryst.* C**69**, 1144–1146.

Parsons, S., Smith, A. A. & Winpenny, R. E. P. (2000). *Chem. Commun.* 579–580.

Samolová, E. & Fábry, J. (2020). *Acta Cryst.* E**76**, 1684–1688.

Schmitz, S., Monakhov, K. Yu., van Leusen, J., Izarova, N. V., Heß, V. & Kögerler, P. (2016). *RSC Adv.* **6**, 100664–100669.

Schmitz, S., Secker, T., Batool, M., van Leusen, J., Nadeem, M. A. & Kögerler, P. (2018). *Inorg. Chim. Acta*, **482**, 522–525.

Sheldrick, G. M. (2015). *Acta Cryst.* C**71**, 3–8.

Stoe & Cie GmbH, *X_AREA, X-RED32, X-SHAPE and LANA.* (2019). Stoe & Cie, Darmstadt, Germany.

Figure 1

ORTEP plot of the monomeric unit of (**I**) with thermal ellipsoids at the 30 % probability level for all non-H atoms. H-atoms are omitted for clarity. Color code: Sr teal, C (ib) gray, C (H_2mda) green, N blue, O red. Disordered atoms are omitted for clarity. Symmetry codes: (i) 2 – x, 1 – y, 1 – z; (ii) 1 – x, 1 – y, 1 – z.

Figure 2

Representation of a polyhedron around a central Sr ion spanned by the NO_8 coordination environment. Color code: Sr teal, N blue, O red, polyhedron borders black and polyhedron faces transparent.

Figure 3

Representation of a segment of the polymeric structure of $[Sr(ib)_2(H_2mda)]_n$ (**I**) along the crystallographic *c* axis. Color code: Sr teal, C (ib) gray, C (H_2mda) green, N blue, O red, bridging O spheres red and H atoms white. Hydrogen bonds are shown as dashed black lines. Disordered fragments are omitted for clarity.

Figure 4

Packing diagram of (**I**), viewed down the *a* axis (left) and the *c* axis (right). Color code: Sr teal, C (ib) gray, C (H_2mda) green, N blue, O red. H atoms and disordered fragments are omitted for clarity.

Figure 5

Synthesis of compound **I**.

Figure 6

Thermogravimetric analysis for **I**.

supporting information

Synthesis and crystal structure of a one-dimensional chain-like strontium(II) coordination polymer built of *N*-methyldiethanolamine and isobutyrate ligands

Maximilian Seiß, Sebastian Schmitz, Martin Börner and Kirill Yu. Monakhov*

Computing details

Data collection: *X-AREA* Pilatus3_SV 1.31.154.0 (STOE, 2019); cell refinement: *X-AREA* Recipe 1.34.0.0 (STOE, 2019); data reduction: *X-AREA* Integrate 1.77.0.0 (STOE, 2019) *X-AREA* LANA 1.77.1.0 (STOE, 2019); program(s) used to solve structure: olex2.solve 1.3 (Bourhis *et al.*, 2015); program(s) used to refine structure: *SHELXL* 2018/3 (Sheldrick, 2015); molecular graphics: Olex2 1.3 (Dolomanov *et al.*, 2009); software used to prepare material for publication: STOE & Cie GmbH, *X-AREA*, software package for collecting single-crystal or multi-domain crystal data on STOE area-detector diffractometers, for image processing, for the correction and scaling of reflection intensities and for outlier rejection, version 1.88, Darmstadt 2019.

[Sr(ib)2(H2mda)]n

Crystal data

$C_{13}H_{27}NO_6Sr$	$F(000) = 792$
$M_r = 380.97$	$D_x = 1.434$ Mg m^{-3}
Monoclinic, $P2_1/c$	Cu $K\alpha$ radiation, $\lambda = 1.54186$ Å
$a = 8.1516$ (2) Å	Cell parameters from 16191 reflections
$b = 19.1921$ (6) Å	$\theta = 4.5–70.9°$
$c = 11.4288$ (3) Å	$\mu = 4.46$ mm^{-1}
$\beta = 99.295$ (2)°	$T = 180$ K
$V = 1764.52$ (8) Å^3	Block, light yellow
$Z = 4$	0.28 × 0.21 × 0.13 mm

Data collection

STOE STADIVARI diffractometer	Absorption correction: multi-scan STOE LANA, absorption correction by scaling of reflection intensities. J. Koziskova, F. Hahn, J. Richter, J. Kozisek, "Comparison of different absorption corrections on the model structure of tetrakis(μ_2-acetato)- diaqua-di-copper(II)", Acta Chimica Slovaca, vol. 9, no. 2, 2016, pp. 136 - 140.
Radiation source: GeniX 3D HF Cu	$T_{min} = 0.178$, $T_{max} = 0.458$
Graded multilayer mirror monochromator	15496 measured reflections
Detector resolution: 5.81 pixels mm^{-1}	3300 independent reflections
rotation method, ω scans	3009 reflections with $I > 2\sigma(I)$
	$R_{int} = 0.014$
	$\theta_{max} = 70.5°$, $\theta_{min} = 4.6°$
	$h = -6 \rightarrow 9$
	$k = -23 \rightarrow 22$
	$l = -13 \rightarrow 13$

Refinement

Refinement on F^2
Least-squares matrix: full
$R[F^2 > 2\sigma(F^2)] = 0.018$
$wR(F^2) = 0.046$
$S = 1.06$
3300 reflections
240 parameters
0 restraints
Primary atom site location: iterative
Hydrogen site location: inferred from neighbouring sites
H-atom parameters constrained
$w = 1/[\sigma^2(F_o^2) + (0.031P)^2 + 0.1664P]$ where $P = (F_o^2 + 2F_c^2)/3$
$(\Delta/\sigma)_{max} = 0.002$
$\Delta\rho_{max} = 0.39$ e Å^{-3}
$\Delta\rho_{min} = -0.19$ e Å^{-3}

Special details

Geometry. All esds (except the esd in the dihedral angle between two l.s. planes) are estimated using the full covariance matrix. The cell esds are taken into account individually in the estimation of esds in distances, angles and torsion angles; correlations between esds in cell parameters are only used when they are defined by crystal symmetry. An approximate (isotropic) treatment of cell esds is used for estimating esds involving l.s. planes.

Fractional atomic coordinates and isotropic or equivalent isotropic displacement parameters (Å^2) for [Sr(ib)2(H2mda)]n

	x	*y*	*z*	U_{iso}*/U_{eq}	Occ. (<1)
Sr1	0.74277 (2)	0.53023 (2)	0.46969 (2)	0.02003 (5)	
O1	1.03404 (12)	0.57713 (5)	0.53587 (9)	0.0276 (2)	
N1	0.71783 (17)	0.65620 (7)	0.33604 (12)	0.0315 (3)	
C1	1.14299 (18)	0.61161 (8)	0.60142 (13)	0.0248 (3)	
O2	1.29545 (13)	0.59817 (6)	0.60970 (11)	0.0350 (3)	
C2	1.0907 (2)	0.66959 (9)	0.67912 (16)	0.0333 (4)	
H2	0.967393	0.675584	0.660022	0.040*	
O3	0.44290 (13)	0.51634 (6)	0.36832 (9)	0.0277 (2)	
C3	1.1366 (3)	0.64916 (12)	0.80933 (18)	0.0564 (6)	
H3A	1.079880	0.605721	0.823650	0.085*	
H3B	1.102401	0.686203	0.859261	0.085*	
H3C	1.257147	0.642439	0.828695	0.085*	
O4	0.18685 (13)	0.48389 (7)	0.29816 (10)	0.0360 (3)	
C4	1.1738 (3)	0.73767 (10)	0.6536 (2)	0.0579 (6)	
H4A	1.294585	0.731263	0.665716	0.087*	
H4B	1.145448	0.773973	0.707215	0.087*	
H4C	1.134732	0.751626	0.571288	0.087*	
O5A	0.577 (3)	0.6350 (9)	0.5311 (11)	0.0265 (9)	0.381 (3)
H5A	0.609072	0.636661	0.604661	0.040*	0.381 (3)
C5	0.33403 (18)	0.49642 (8)	0.28442 (13)	0.0248 (3)	
O6A	0.882 (6)	0.530 (3)	0.273 (5)	0.0294 (17)	0.381 (3)
H6A	0.964326	0.504679	0.267529	0.044*	0.381 (3)
C6	0.3829 (2)	0.48362 (10)	0.16298 (15)	0.0350 (4)	
H6	0.502060	0.497040	0.166344	0.042*	
C7	0.2768 (3)	0.52822 (12)	0.06894 (18)	0.0542 (6)	
H7A	0.159415	0.515843	0.065063	0.081*	
H7B	0.310497	0.519856	−0.008377	0.081*	
H7C	0.292495	0.577568	0.089822	0.081*	
C8	0.3644 (3)	0.40680 (11)	0.13232 (17)	0.0484 (5)	
H8A	0.432387	0.379290	0.194290	0.073*	
H8B	0.401459	0.398322	0.056146	0.073*	
H8C	0.247570	0.393170	0.126731	0.073*	
C9A	0.5616 (6)	0.6538 (3)	0.2356 (5)	0.0484 (14)	0.381 (3)

H9AA	0.462861	0.641832	0.270231	0.073*	0.381 (3)
H9AB	0.578748	0.618626	0.176647	0.073*	0.381 (3)
H9AC	0.545747	0.699558	0.197278	0.073*	0.381 (3)
C10A	0.6919 (7)	0.7102 (2)	0.4143 (5)	0.0447 (13)	0.381 (3)
H10A	0.658994	0.752626	0.367158	0.054*	0.381 (3)
H10B	0.798802	0.720209	0.466068	0.054*	0.381 (3)
C11A	0.5675 (15)	0.6966 (8)	0.4890 (14)	0.0384 (12)	0.381 (3)
H11A	0.581608	0.730392	0.555314	0.046*	0.381 (3)
H11B	0.455628	0.703709	0.442146	0.046*	0.381 (3)
C12A	0.8593 (6)	0.6604 (2)	0.2824 (4)	0.0372 (12)	0.381 (3)
H12A	0.957084	0.667164	0.345023	0.045*	0.381 (3)
H12B	0.849687	0.702121	0.230877	0.045*	0.381 (3)
C13A	0.893 (4)	0.5941 (18)	0.205 (3)	0.0431 (8)	0.381 (3)
H13A	0.809510	0.592742	0.131210	0.052*	0.381 (3)
H13B	1.004460	0.597745	0.181722	0.052*	0.381 (3)
O6	0.866 (3)	0.5342 (16)	0.268 (3)	0.0294 (17)	0.619 (3)
H6B	0.960128	0.517184	0.294366	0.044*	0.619 (3)
O5	0.5875 (15)	0.6406 (5)	0.5558 (6)	0.0265 (9)	0.619 (3)
H5	0.505903	0.619209	0.574990	0.040*	0.619 (3)
C9	0.8519 (4)	0.70964 (15)	0.3830 (3)	0.0411 (7)	0.619 (3)
H9A	0.961638	0.690803	0.376032	0.062*	0.619 (3)
H9B	0.846963	0.719638	0.466490	0.062*	0.619 (3)
H9C	0.832910	0.752678	0.336686	0.062*	0.619 (3)
C11	0.5250 (7)	0.6997 (5)	0.4694 (8)	0.0384 (12)	0.619 (3)
H11C	0.403861	0.705598	0.467052	0.046*	0.619 (3)
H11D	0.580042	0.743736	0.498683	0.046*	0.619 (3)
C12	0.7389 (4)	0.63928 (16)	0.2165 (2)	0.0404 (8)	0.619 (3)
H12C	0.747000	0.683090	0.172108	0.048*	0.619 (3)
H12D	0.639044	0.613961	0.177495	0.048*	0.619 (3)
C13	0.887 (2)	0.5965 (11)	0.2096 (17)	0.0431 (8)	0.619 (3)
H13C	0.896781	0.587366	0.125768	0.052*	0.619 (3)
H13D	0.988442	0.620832	0.247982	0.052*	0.619 (3)
C10	0.5591 (3)	0.68558 (14)	0.3441 (3)	0.0372 (7)	0.619 (3)
H10C	0.472220	0.653633	0.304369	0.045*	0.619 (3)
H10D	0.548179	0.730028	0.299556	0.045*	0.619 (3)

Atomic displacement parameters (Å^2) for [Sr(ib)2(H2mda)]n

	U^{11}	U^{22}	U^{33}	U^{12}	U^{13}	U^{23}
Sr1	0.01411 (8)	0.02466 (8)	0.02156 (8)	−0.00039 (4)	0.00361 (5)	−0.00108 (5)
O1	0.0218 (5)	0.0283 (5)	0.0319 (6)	−0.0029 (4)	0.0016 (4)	−0.0046 (4)
N1	0.0321 (8)	0.0296 (7)	0.0338 (7)	0.0040 (5)	0.0082 (6)	0.0048 (6)
C1	0.0201 (7)	0.0262 (7)	0.0287 (7)	−0.0014 (5)	0.0059 (5)	−0.0009 (6)
O2	0.0176 (6)	0.0348 (6)	0.0537 (7)	−0.0017 (4)	0.0092 (5)	−0.0128 (5)
C2	0.0233 (8)	0.0343 (8)	0.0418 (9)	0.0036 (6)	0.0040 (6)	−0.0114 (7)
O3	0.0203 (5)	0.0360 (6)	0.0259 (5)	−0.0021 (4)	0.0010 (4)	−0.0006 (4)
C3	0.0662 (14)	0.0652 (14)	0.0378 (11)	0.0156 (11)	0.0084 (9)	−0.0165 (10)
O4	0.0177 (6)	0.0627 (8)	0.0276 (6)	−0.0007 (5)	0.0043 (4)	0.0012 (5)
C4	0.0619 (14)	0.0340 (10)	0.0818 (16)	−0.0043 (9)	0.0239 (11)	−0.0198 (10)
O5A	0.0273 (17)	0.0322 (16)	0.021 (3)	−0.0033 (11)	0.008 (3)	−0.0085 (19)
C5	0.0200 (8)	0.0308 (8)	0.0235 (7)	0.0022 (6)	0.0031 (5)	0.0009 (6)

O6A	0.023 (4)	0.035 (3)	0.031 (2)	0.007 (3)	0.007 (3)	0.0037 (18)
C6	0.0265 (9)	0.0539 (10)	0.0260 (8)	−0.0021 (7)	0.0088 (6)	−0.0036 (7)
C7	0.0684 (15)	0.0683 (14)	0.0276 (9)	0.0088 (11)	0.0131 (9)	0.0082 (9)
C8	0.0536 (12)	0.0571 (12)	0.0346 (10)	0.0073 (9)	0.0073 (8)	−0.0139 (9)
C9A	0.037 (3)	0.052 (3)	0.052 (3)	−0.001 (2)	−0.005 (2)	0.017 (2)
C10A	0.057 (3)	0.026 (2)	0.056 (3)	−0.003 (2)	0.022 (2)	−0.002 (2)
C11A	0.030 (3)	0.0315 (12)	0.056 (3)	0.017 (2)	0.015 (3)	0.0070 (18)
C12A	0.039 (3)	0.034 (2)	0.042 (3)	0.0017 (18)	0.015 (2)	0.015 (2)
C13A	0.0521 (15)	0.0471 (17)	0.0352 (18)	0.0102 (11)	0.0227 (11)	0.0109 (12)
O6	0.023 (4)	0.035 (3)	0.031 (2)	0.007 (3)	0.007 (3)	0.0037 (18)
O5	0.0273 (17)	0.0322 (16)	0.021 (3)	−0.0033 (11)	0.008 (3)	−0.0085 (19)
C9	0.0362 (16)	0.0362 (15)	0.0495 (18)	−0.0081 (12)	0.0024 (12)	0.0055 (13)
C11	0.030 (3)	0.0315 (12)	0.056 (3)	0.017 (2)	0.015 (3)	0.0070 (18)
C12	0.051 (2)	0.0420 (16)	0.0275 (14)	0.0089 (13)	0.0056 (12)	0.0089 (12)
C13	0.0521 (15)	0.0471 (17)	0.0352 (18)	0.0102 (11)	0.0227 (11)	0.0109 (12)
C10	0.0311 (15)	0.0330 (14)	0.0478 (17)	0.0068 (11)	0.0076 (12)	0.0106 (12)

Geometric parameters (Å, º) for [Sr(ib)2(H2mda)]n

Sr1—O1	2.5377 (10)	C6—C7	1.528 (3)
Sr1—O1^{i}	2.7563 (10)	C6—C8	1.517 (3)
Sr1—N1	2.8495 (13)	C7—H7A	0.9800
Sr1—O2^{i}	2.6270 (11)	C7—H7B	0.9800
Sr1—O3^{ii}	2.7244 (11)	C7—H7C	0.9800
Sr1—O3	2.5444 (10)	C8—H8A	0.9800
Sr1—O4^{ii}	2.6364 (11)	C8—H8B	0.9800
Sr1—O5A	2.58 (2)	C8—H8C	0.9800
Sr1—C5^{ii}	3.0199 (15)	C9A—H9AA	0.9800
Sr1—O6A	2.68 (6)	C9A—H9AB	0.9800
Sr1—O6	2.66 (3)	C9A—H9AC	0.9800
Sr1—O5	2.731 (11)	C10A—H10A	0.9900
O1—C1	1.2541 (18)	C10A—H10B	0.9900
N1—C9A	1.571 (5)	C10A—C11A	1.450 (16)
N1—C10A	1.407 (5)	C11A—H11A	0.9900
N1—C12A	1.393 (5)	C11A—H11B	0.9900
N1—C9	1.531 (3)	C12A—H12A	0.9900
N1—C12	1.441 (3)	C12A—H12B	0.9900
N1—C10	1.428 (3)	C12A—C13A	1.60 (3)
C1—O2	1.2578 (19)	C13A—H13A	0.9900
C1—C2	1.527 (2)	C13A—H13B	0.9900
C2—H2	1.0000	O6—H6B	0.8400
C2—C3	1.526 (3)	O6—C13	1.39 (4)
C2—C4	1.521 (3)	O5—H5	0.8400
O3—C5	1.2563 (18)	O5—C11	1.537 (12)
C3—H3A	0.9800	C9—H9A	0.9800
C3—H3B	0.9800	C9—H9B	0.9800
C3—H3C	0.9800	C9—H9C	0.9800
O4—C5	1.2581 (19)	C11—H11C	0.9900
C4—H4A	0.9800	C11—H11D	0.9900
C4—H4B	0.9800	C11—C10	1.526 (10)
C4—H4C	0.9800	C12—H12C	0.9900

O5A—H5A	0.8400	C12—H12D	0.9900
O5A—C11A	1.27 (2)	C12—C13	1.471 (18)
C5—C6	1.524 (2)	C13—H13C	0.9900
O6A—H6A	0.8400	C13—H13D	0.9900
O6A—C13A	1.46 (6)	C10—H10C	0.9900
C6—H6	1.0000	C10—H10D	0.9900

O1—Sr1—O1^{i}	71.51 (4)	H4A—C4—H4C	109.5
O1—Sr1—N1	80.86 (4)	H4B—C4—H4C	109.5
O1^{i}—Sr1—N1	127.74 (4)	Sr1—O5A—H5A	102.1
O1—Sr1—O2^{i}	119.26 (3)	C11A—O5A—Sr1	128.5 (14)
O1—Sr1—O3	163.08 (4)	C11A—O5A—H5A	109.5
O1—Sr1—O3^{ii}	120.63 (3)	O3—C5—Sr1^{ii}	64.42 (8)
O1—Sr1—O4^{ii}	72.27 (3)	O3—C5—O4	122.22 (14)
O1—Sr1—O5A	98.5 (4)	O3—C5—C6	119.20 (14)
O1^{i}—Sr1—C5^{ii}	97.57 (4)	O4—C5—Sr1^{ii}	60.41 (8)
O1—Sr1—C5^{ii}	96.16 (4)	O4—C5—C6	118.51 (13)
O1—Sr1—O6A	75.4 (11)	C6—C5—Sr1^{ii}	160.72 (11)
O1—Sr1—O6	77.3 (6)	Sr1—O6A—H6A	120.7
O1—Sr1—O5	94.8 (2)	C13A—O6A—Sr1	121 (3)
N1—Sr1—C5^{ii}	129.42 (4)	C13A—O6A—H6A	109.5
O2^{i}—Sr1—O1^{i}	48.14 (3)	C5—C6—H6	108.6
O2^{i}—Sr1—N1	128.06 (4)	C5—C6—C7	110.42 (15)
O2^{i}—Sr1—O3^{ii}	83.06 (4)	C7—C6—H6	108.6
O2^{i}—Sr1—O4^{ii}	104.22 (4)	C8—C6—C5	109.65 (15)
O2^{i}—Sr1—C5^{ii}	97.57 (4)	C8—C6—H6	108.6
O2^{i}—Sr1—O6A	75.6 (12)	C8—C6—C7	110.99 (16)
O2^{i}—Sr1—O6	76.6 (7)	C6—C7—H7A	109.5
O2^{i}—Sr1—O5	144.7 (2)	C6—C7—H7B	109.5
O3—Sr1—O1^{i}	120.02 (3)	C6—C7—H7C	109.5
O3^{ii}—Sr1—O1^{i}	102.22 (3)	H7A—C7—H7B	109.5
O3—Sr1—N1	82.22 (4)	H7A—C7—H7C	109.5
O3^{ii}—Sr1—N1	130.04 (4)	H7B—C7—H7C	109.5
O3—Sr1—O2^{i}	72.11 (3)	C6—C8—H8A	109.5
O3—Sr1—O3^{ii}	71.14 (4)	C6—C8—H8B	109.5
O3—Sr1—O4^{ii}	118.97 (3)	C6—C8—H8C	109.5
O3—Sr1—O5A	72.5 (4)	H8A—C8—H8B	109.5
O3—Sr1—C5^{ii}	94.46 (4)	H8A—C8—H8C	109.5
O3^{ii}—Sr1—C5^{ii}	24.58 (4)	H8B—C8—H8C	109.5
O3—Sr1—O6A	97.1 (10)	N1—C9A—H9AA	109.5
O3—Sr1—O6	94.5 (6)	N1—C9A—H9AB	109.5
O3^{ii}—Sr1—O5	70.7 (2)	N1—C9A—H9AC	109.5
O3—Sr1—O5	77.2 (2)	H9AA—C9A—H9AB	109.5
O4^{ii}—Sr1—O1^{i}	84.80 (3)	H9AA—C9A—H9AC	109.5
O4^{ii}—Sr1—N1	127.72 (4)	H9AB—C9A—H9AC	109.5
O4^{ii}—Sr1—O3^{ii}	48.46 (3)	N1—C10A—H10A	108.4
O4^{ii}—Sr1—C5^{ii}	24.52 (4)	N1—C10A—H10B	108.4
O4^{ii}—Sr1—O6A	142.4 (9)	N1—C10A—C11A	115.7 (8)
O4^{ii}—Sr1—O6	145.4 (6)	H10A—C10A—H10B	107.4
O4^{ii}—Sr1—O5	75.82 (16)	C11A—C10A—H10A	108.4
O5A—Sr1—N1	59.3 (4)	C11A—C10A—H10B	108.4

O5A—Sr1—C5^{ii}	71.5 (4)	O5A—C11A—C10A	112.5 (14)
O5A—Sr1—O6A	122.7 (11)	O5A—C11A—H11A	109.1
O6A—Sr1—N1	63.5 (10)	O5A—C11A—H11B	109.1
O6A—Sr1—C5^{ii}	164.0 (9)	C10A—C11A—H11A	109.1
O6—Sr1—O1^{i}	70.0 (6)	C10A—C11A—H11B	109.1
O6—Sr1—N1	61.0 (6)	H11A—C11A—H11B	107.8
O6—Sr1—O3^{ii}	158.1 (7)	N1—C12A—H12A	108.5
O6—Sr1—C5^{ii}	167.2 (5)	N1—C12A—H12B	108.5
O6—Sr1—O5	123.5 (7)	N1—C12A—C13A	115.2 (12)
O5—Sr1—O1^{i}	159.08 (16)	H12A—C12A—H12B	107.5
O5—Sr1—N1	62.5 (2)	C13A—C12A—H12A	108.5
O5—Sr1—C5^{ii}	67.5 (2)	C13A—C12A—H12B	108.5
Sr1—O1—Sr1^{i}	108.50 (4)	O6A—C13A—C12A	110 (3)
C1—O1—Sr1	154.61 (10)	O6A—C13A—H13A	109.7
C1—O1—Sr1^{i}	90.10 (9)	O6A—C13A—H13B	109.7
C9A—N1—Sr1	110.3 (2)	C12A—C13A—H13A	109.7
C10A—N1—Sr1	106.8 (2)	C12A—C13A—H13B	109.7
C10A—N1—C9A	107.4 (3)	H13A—C13A—H13B	108.2
C12A—N1—Sr1	106.99 (19)	Sr1—O6—H6B	97.5
C12A—N1—C9A	108.1 (3)	C13—O6—Sr1	122 (2)
C12A—N1—C10A	117.2 (3)	C13—O6—H6B	109.5
C9—N1—Sr1	113.27 (13)	Sr1—O5—H5	98.5
C12—N1—Sr1	107.93 (14)	C11—O5—Sr1	117.7 (5)
C12—N1—C9	107.2 (2)	C11—O5—H5	109.5
C10—N1—Sr1	106.71 (13)	N1—C9—H9A	109.5
C10—N1—C9	108.39 (19)	N1—C9—H9B	109.5
C10—N1—C12	113.45 (19)	N1—C9—H9C	109.5
O1—C1—Sr1^{i}	65.45 (8)	H9A—C9—H9B	109.5
O1—C1—O2	122.18 (14)	H9A—C9—H9C	109.5
O1—C1—C2	119.64 (13)	H9B—C9—H9C	109.5
O2—C1—Sr1^{i}	59.55 (8)	O5—C11—H11C	109.1
O2—C1—C2	118.10 (13)	O5—C11—H11D	109.1
C2—C1—Sr1^{i}	159.64 (11)	H11C—C11—H11D	107.9
C1—O2—Sr1^{i}	96.07 (9)	C10—C11—O5	112.4 (6)
C1—C2—H2	108.8	C10—C11—H11C	109.1
C3—C2—C1	109.41 (14)	C10—C11—H11D	109.1
C3—C2—H2	108.8	N1—C12—H12C	108.8
C4—C2—C1	109.84 (15)	N1—C12—H12D	108.8
C4—C2—H2	108.8	N1—C12—C13	113.6 (8)
C4—C2—C3	111.17 (17)	H12C—C12—H12D	107.7
Sr1—O3—Sr1^{ii}	108.86 (4)	C13—C12—H12C	108.8
C5—O3—Sr1^{ii}	91.00 (9)	C13—C12—H12D	108.8
C5—O3—Sr1	152.58 (10)	O6—C13—C12	106.9 (17)
C2—C3—H3A	109.5	O6—C13—H13C	110.3
C2—C3—H3B	109.5	O6—C13—H13D	110.3
C2—C3—H3C	109.5	C12—C13—H13C	110.3
H3A—C3—H3B	109.5	C12—C13—H13D	110.3
H3A—C3—H3C	109.5	H13C—C13—H13D	108.6
H3B—C3—H3C	109.5	N1—C10—C11	115.8 (3)
C5—O4—Sr1^{ii}	95.07 (9)	N1—C10—H10C	108.3
C2—C4—H4A	109.5	N1—C10—H10D	108.3

C2—C4—H4B	109.5	C11—C10—H10C	108.3
C2—C4—H4C	109.5	C11—C10—H10D	108.3
H4A—C4—H4B	109.5	H10C—C10—H10D	107.4
Sr1—O1—C1—Sr1^{i}	138.0 (2)	Sr1—O5—C11—C10	1.0 (9)
Sr1^{i}—O1—C1—O2	18.98 (15)	O1—C1—O2—Sr1^{i}	−20.07 (16)
Sr1—O1—C1—O2	157.02 (16)	O1—C1—C2—C3	113.07 (18)
Sr1^{i}—O1—C1—C2	−157.82 (13)	O1—C1—C2—C4	−124.64 (17)
Sr1—O1—C1—C2	−19.8 (3)	N1—C10A—C11A—O5A	42.3 (14)
Sr1—N1—C10A—C11A	−46.2 (7)	N1—C12A—C13A—O6A	−49 (3)
Sr1—N1—C12A—C13A	54.3 (13)	N1—C12—C13—O6	61.3 (18)
Sr1—N1—C12—C13	−50.4 (9)	O2—C1—C2—C3	−63.9 (2)
Sr1—N1—C10—C11	57.0 (5)	O2—C1—C2—C4	58.4 (2)
Sr1^{i}—C1—C2—C3	13.9 (4)	C2—C1—O2—Sr1^{i}	156.78 (12)
Sr1^{i}—C1—C2—C4	136.2 (3)	O3—C5—C6—C7	−123.02 (18)
Sr1—O3—C5—Sr1^{ii}	137.4 (2)	O3—C5—C6—C8	114.37 (17)
Sr1^{ii}—O3—C5—O4	18.43 (16)	O4—C5—C6—C7	59.8 (2)
Sr1—O3—C5—O4	155.79 (15)	O4—C5—C6—C8	−62.8 (2)
Sr1^{ii}—O3—C5—C6	−158.64 (13)	C9A—N1—C10A—C11A	72.0 (7)
Sr1—O3—C5—C6	−21.3 (3)	C9A—N1—C12A—C13A	−64.4 (13)
Sr1^{ii}—O4—C5—O3	−19.14 (16)	C10A—N1—C12A—C13A	174.1 (13)
Sr1^{ii}—O4—C5—C6	157.94 (13)	C12A—N1—C10A—C11A	−166.1 (6)
Sr1—O5A—C11A—C10A	−12.8 (19)	O5—C11—C10—N1	−41.3 (8)
Sr1^{ii}—C5—C6—C7	141.2 (3)	C9—N1—C12—C13	72.0 (9)
Sr1^{ii}—C5—C6—C8	18.6 (4)	C9—N1—C10—C11	−65.3 (5)
Sr1—O6A—C13A—C12A	15 (4)	C12—N1—C10—C11	175.7 (5)
Sr1—O6—C13—C12	−41 (2)	C10—N1—C12—C13	−168.4 (9)

Symmetry codes: (i) −*x*+2, −*y*+1, −*z*+1; (ii) −*x*+1, −*y*+1, −*z*+1.

Hydrogen-bond geometry (Å, º) for [Sr(ib)2(H2mda)]n

D—H···*A*	*D*—H	H···*A*	*D*···*A*	*D*—H···*A*
O6*A*—H6*A*···O4^{iii}	0.84	1.83	2.61 (5)	153
O6—H6*B*···O4^{iii}	0.84	1.95	2.75 (3)	160
O5—H5···O2^{iv}	0.84	1.87	2.680 (12)	163

Symmetry codes: (iii) *x*+1, *y*, *z*; (iv) *x*−1, *y*, *z*.

Selected bond lengths (Å) and angles (°).

Sr1 – O1^{i}	2.7568 (13)	Sr1 – O6	2.655 (4)
Sr1 – O3^{ii}	2.7251 (14)	Sr1 – N1	2.8516 (16)
Sr1 – O1	2.5370 (12)	Sr1 ··· Sr1^{i}	4.2981 (3)
Sr1 – O3	2.5441 (13)	Sr1 ··· Sr1^{ii}	4.2868 (3)
Sr1 – O2^{i}	2.6273 (14)	Sr1 – O1 – Sr1^{i}	108.50 (4)
Sr1 – O4^{ii}	2.6367 (14)	Sr1 – O1 – Sr1^{ii}	108.84 (5)
Sr1 – O5	2.678 (4)		

Symmetry codes: (i) 2 – x, 1 – y, 1 – z; (ii) 1 – x, 1 – y, 1 – z.

Selected Continuous Shapes Measures (CShM) values for the geometry about the nine-coordinate Sr(II) ions of I.

Shape	Capped square antiprism (C_{4v}, J10)	Spherical capped square antiprism (C_{4v})	Tricapped trigonal prism (D_{3h}, J51)	Spherical tricapped trigonal prism (D_{3h})	Muffin (C_s)
Sr^{i}	4.349	3.765	5.892	3.696	3.732
Sr^{ii}	4.026	3.346	5.575	3.423	3.358

Symmetry codes: (i) 2 – x, 1 – y, 1 – z; (ii) 1 – x, 1 – y, 1 – z.
