# Supplementary figures and images for: Synthesis and crystal structure of a one-dimensional chain-like strontium(II) coordination polymer built of N-methyldi­ethano­lamine and isobutyrate ligands
Source: Acta Crystallogr E Crystallogr Commun. 2021 Jun 11;77(Pt 7):703–7. doi: 10.1107/S2056989021005594 (PMC8382055; doi:10.1107/S2056989021005594)

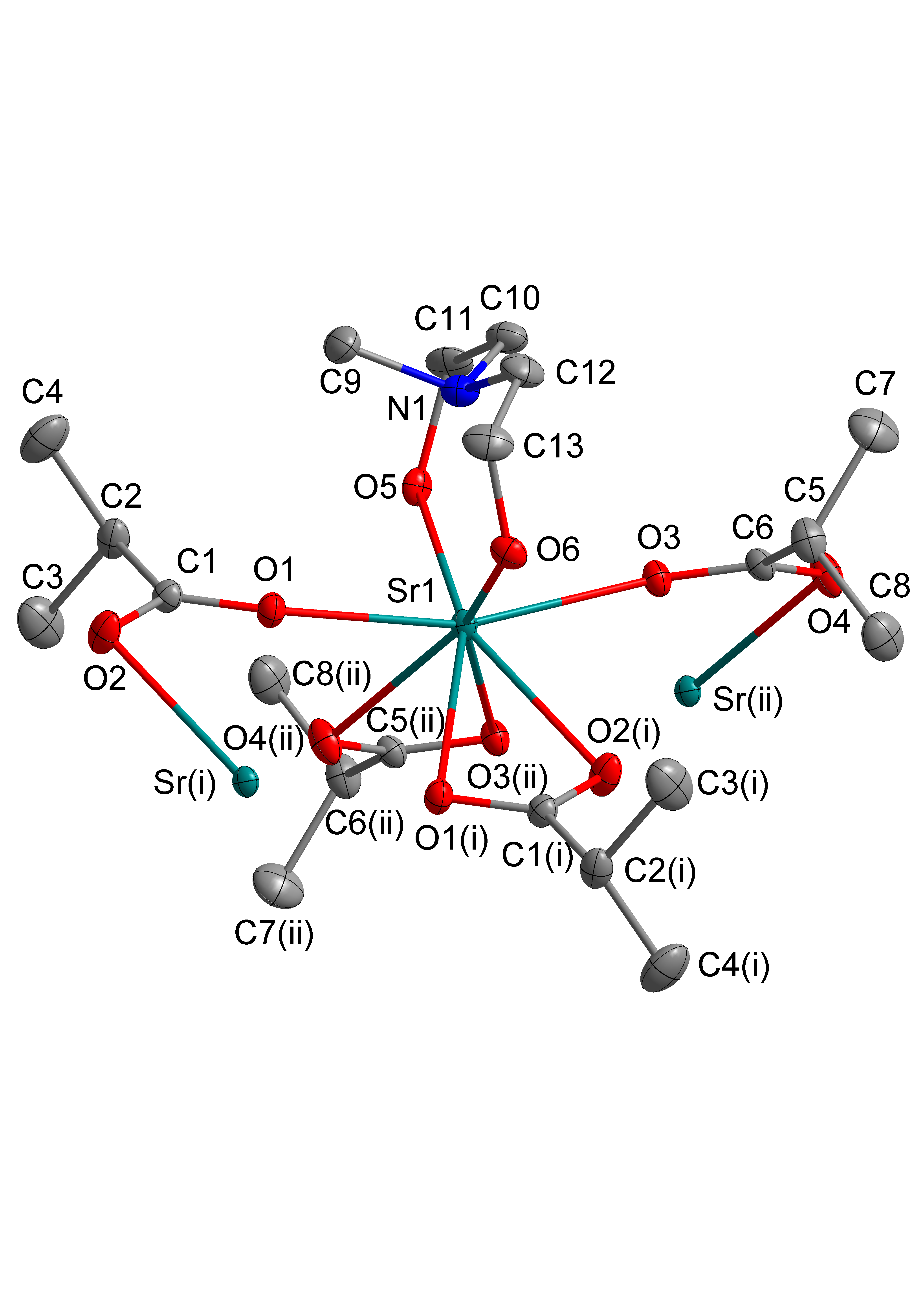

Supplement: Supplementary file 3 [file e-77-00703-sup4.zip › Figure 1.tif]

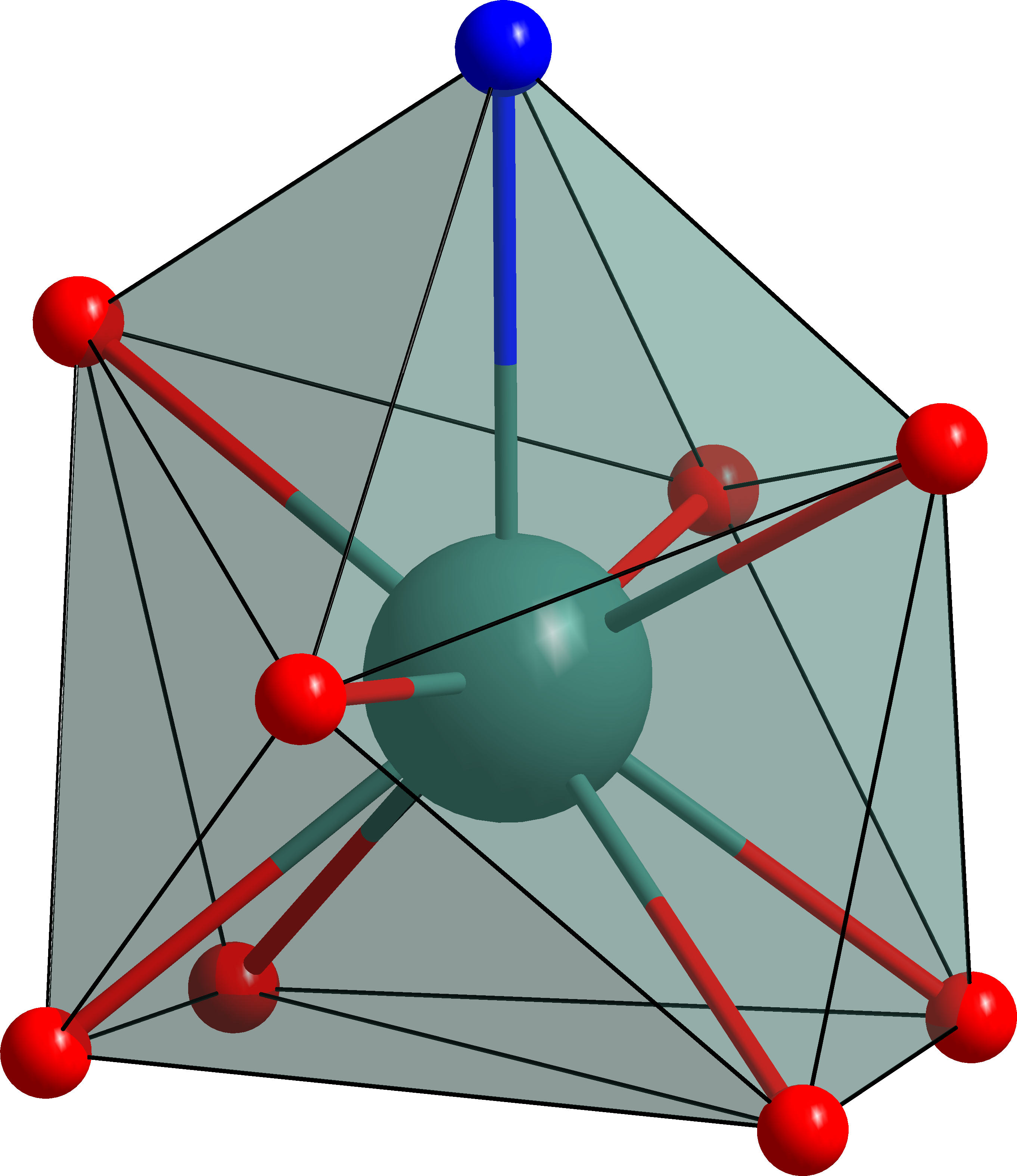

Supplement: Supplementary file 3 [file e-77-00703-sup4.zip › Figure 2.tif]

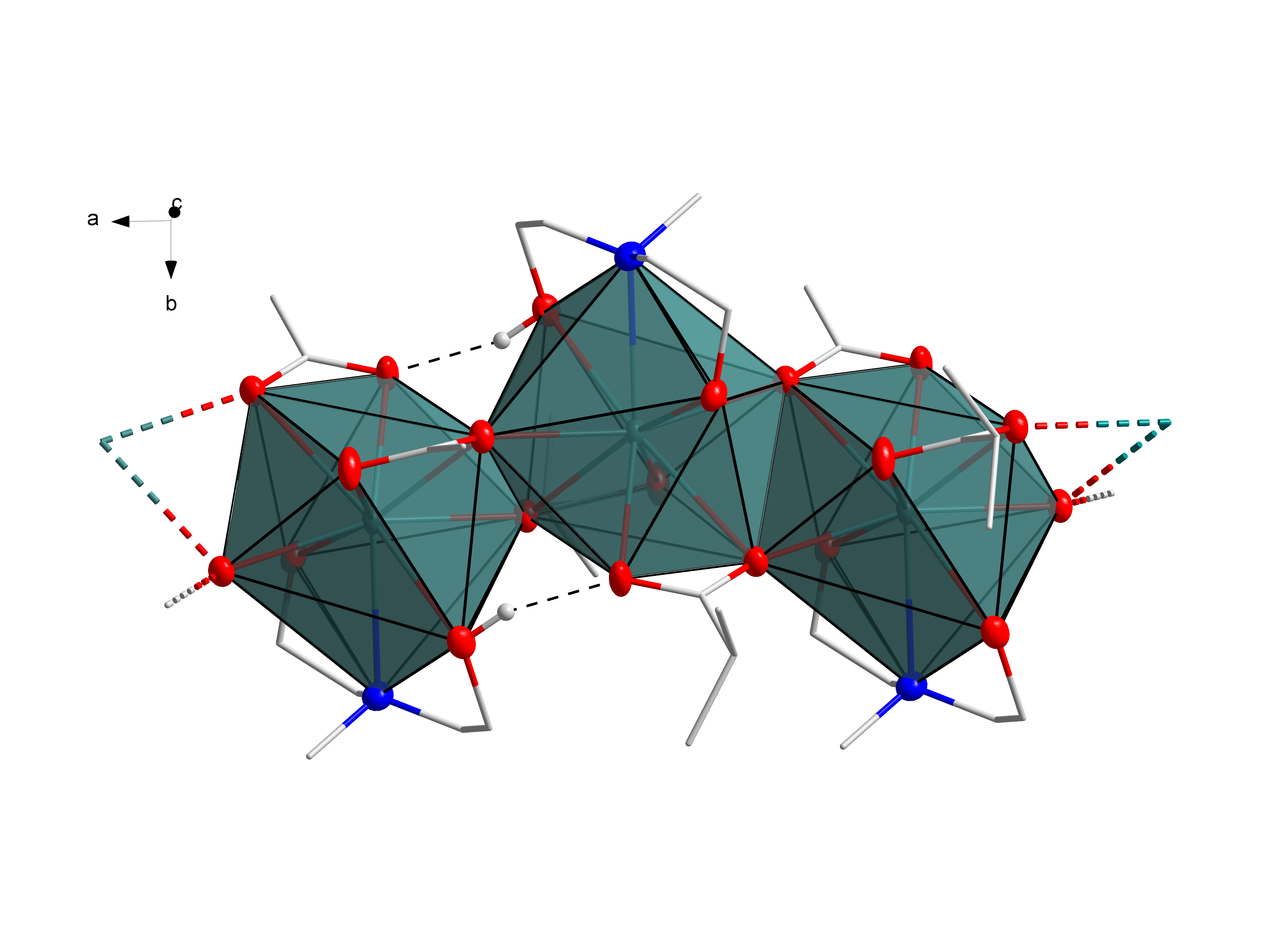

Supplement: Supplementary file 3 [file e-77-00703-sup4.zip › Figure 3.tif]

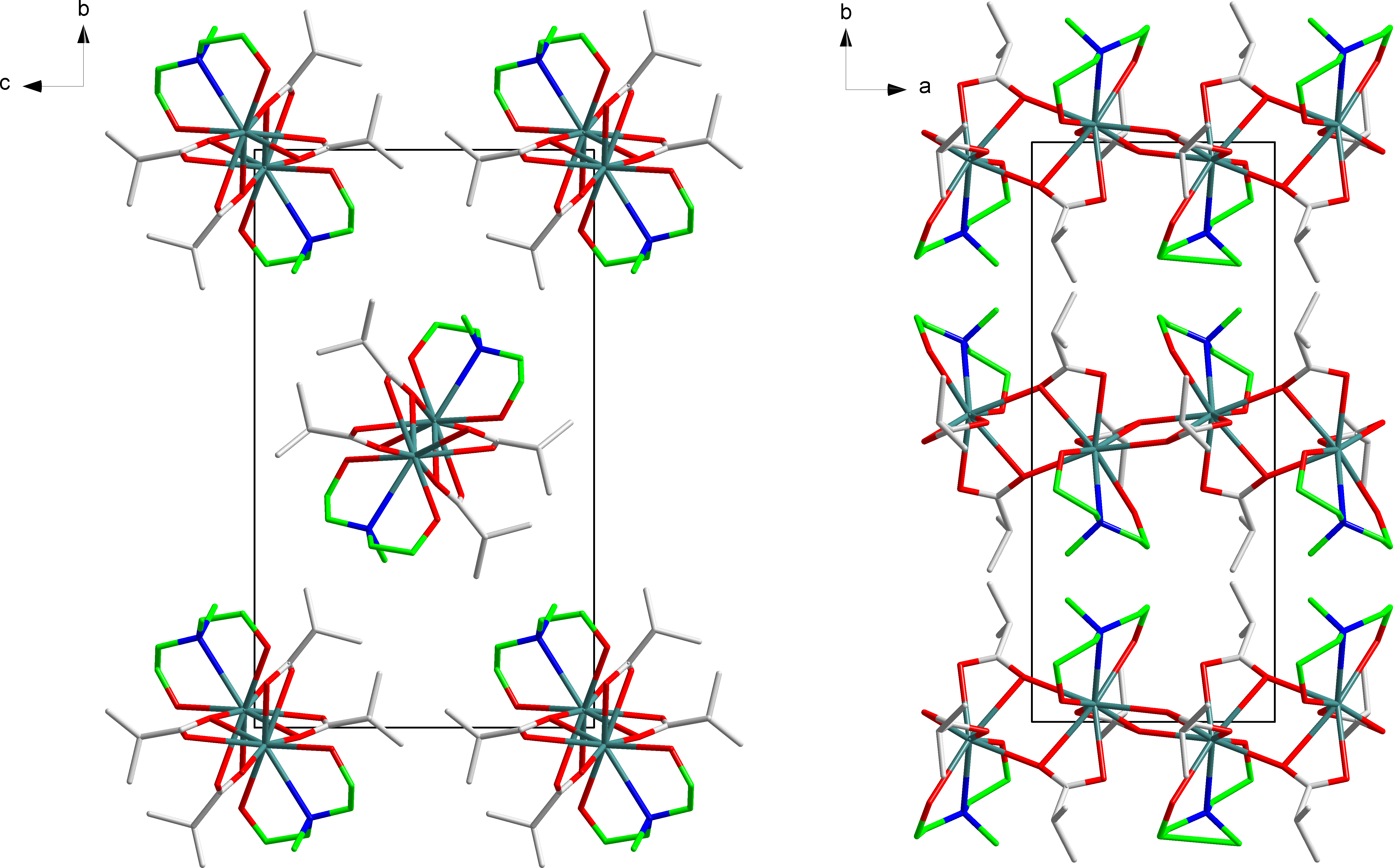

Supplement: Supplementary file 3 [file e-77-00703-sup4.zip › Figure 4.tif]
